# Analysis of physiological characteristics and gene co-expression networks in *Medicago sativa* roots under low-temperature stress

**DOI:** 10.3389/fpls.2025.1597949

**Published:** 2025-08-25

**Authors:** Xiaolong Wang, Hua Chai, Yanxia Xu, Shasha Li, Yue Wu, Ruoding Wang, Zhao Yang

**Affiliations:** Branch of Animal Husbandry and Veterinary of Heilongjiang Academy of Agricultural Sciences, Qiqihar, Heilongjiang, China

**Keywords:** *Medicago sativa*, physiological characteristics, genes, cold tolerance, correlation analysis

## Abstract

*Medicago sativa* is the most widely cultivated high-protein forage crop globally. However, its cultivation in high-latitude and cold regions of China is significantly hindered by low-temperature stress, particularly impacting the root system, the primary functional tissue crucial for winter survival. The physiological and molecular mechanisms underlying the root system’s adaptation and tolerance to low temperatures remain poorly understood. To this end, this study utilized cold-tolerant *Medicago sativa* “Lomgmu801” and the cold-sensitive *Medicago sativa* “Sardi” genotypes as experimental materials to investigate root physiological responses during the overwintering period. Physiological indices, including soluble sugars (SS), proline (Pro), glutathione (GSH), jasmonic acid (JA), abscisic acid (ABA) contents, and peroxidase (POD) activity, were quantified. RNA-seq revealed 743 differentially expressed genes (DEGs) between the cold-tolerant and sensitive genotypes. Subsequently, correlation analysis between DEGs and physiological indices revealed that DEGs in the yellow, blue, and turquoise modules were significantly correlated with the levels of POD, GSH, SS, Pro, JA, and ABA. The core genes were predominantly included in the “MAPK signaling pathway”, “glutathione metabolism”, “plant hormone signal transduction”, “arginine and proline metabolism”, and “phenylpropanoid biosynthesis”. Ultimately, DEGs responsive to low-temperature stress were identified, including *MsGST*, *MsRboh*, *MsPOD*, *MsJAZ*, *MsP5CR*, and *MsPP2C*. By integrating physiological characteristics with cold-tolerance-associated genes, this study elucidates the physiological and molecular mechanisms underlying *Medicago sativa* root adaptation to low temperatures. The RNA-seq data and the core DEGs identified provide valuable theoretical insights and targets for future molecular breeding efforts aimed at enhancing cold tolerance in *Medicago sativa*.

## 1 Introduction

Plants are frequently subjected to various abiotic stress conditions during growth, including low temperature, drought, and salt stress. Low-temperature stress has been reported to cause an annual yield reduction of approximately 40% in temperate crops, whereas extreme cold weather results in crop yield losses ranging from 51% to 82% worldwide (Zhang et al., 2022). In recent years, global climate-related disasters have increased in frequency, with sudden temperature drops, cold waves, and other extreme weather events occurring more frequently in certain regions, resulting in a substantial risk of freezing damage to vegetation (Eom et al., 2022). Low-temperature stress adversely impacts normal plant growth and development, disrupting physiological homeostasis, which, in severe cases, can lead to plant death (Soualiou et al., 2022). Therefore, investigating plant morphological characteristics, physiological responses, and molecular regulatory mechanisms under low-temperature conditions is essential for mitigating the detrimental impacts of extreme cold weather on agriculture. Enhancing crop resilience to low-temperature stress is great significance for ensuring food security in China’s agricultural and pastoral industries (Huang et al., 2022).

Alfalfa (*Medicago sativa*) is not only the most extensively cultivated high-protein forage crop in the world (Wang et al., 2021; Ma et al., 2024), but also the most economically valuable forage species in China (Liu et al., 2021). Heilongjiang Province serves as a major modern livestock production hub in China, where there is substantial demand for high-quality *Medicago sativa* for herbivorous livestock such as dairy and beef cattle. However, due to its high latitude and frigid climate, extreme winter conditions, including low temperatures and cold waves, frequently occur in the province, posing a significant threat to *Medicago sativa* root system survival during the winter. Consequently, winter survival of *Medicago sativa* roots has long been a major technical challenge restricting the development of the *Medicago sativa* industry in Heilongjiang. For successful cultivation in Heilongjiang, *Medicago sativa* varieties must demonstrate high yield, exceptional quality, and strong cold resistance. In recent years, high-yield and high-protein *Medicago sativa* cultivars from the United States, Canada, and Australia have been introduced into the province (Miao et al., 2024; Wang et al., 2024a). However, significant decline in winter survival rates and severe frost damage to the root systems have been observed in the Heilongjiang region after two to three years of cultivation (Mervi et al., 2018). Therefore, the capacity of *Medicago sativa* roots to withstand low temperatures directly determines the plants’ winter survival capacity. The root system, as a vital organ for cold resistance, is essential for nutrient storage, directly affecting the plant’s winter hardiness, disease resistance, and yield potential. When exposed to low-temperature stress, plants undergo a series of physiological adaptations to mitigate cold-induced damage. Osmotic balance is maintained by the accumulation of osmoprotectants such as proline (Pro), soluble sugars (SS), and glutathione (GSH) (Bremer et al., 2017). In addition, the activities of cytoprotective enzymes, such as peroxidase (POD) and glutathione-S-transferase (GST), are augmented to combat oxidative damage caused by the overproduction of reactive oxygen species (ROS), including H_2_O_2_ and O_2_·-, which can severely damage cell membranes (Baxter et al., 2014). Additionally, increased accumulation of abscisic acid (ABA) and jasmonic acid (JA) enhances the cold resilience of the roots (Pu et al., 2024). However, physiological responses alone are insufficient to accurately determine the degree of cold tolerance in *Medicago sativa*. A further in-depth understanding requires an integrated analysis of molecular regulatory mechanisms. Among these, the CBF (C-repeat-binding factor) signaling pathway has been extensively investigated (Lee et al., 2021). Research has demonstrated that the *DREB1s/CBFs* transcription factors can trigger the activation of various cold-responsive (*COR*) genes, thereby enhancing cold resilience in *Medicago sativa* (Kidokoro et al., 2022). Moreover, calcium signaling is integral to cold adaptation processes. As a key Ca^2+^ sensor, calmodulin (CaM) participates in the regulation of CaM-mediated pathways, CNGCs (cyclic nucleotide-gated channels), and the MPK3/4/6 (mitogen-activated protein kinase) signaling module. These components positively regulate the ICE-CBF-COR pathway, where *CBF* binds to *COR* gene promoters, inducing *COR* gene expression and ultimately enhancing plant cold resistance (Devireddy et al., 2021; Adhikari et al., 2021; Li et al., 2017; Ding and Yang, 2022). It can thus be concluded that cold tolerance in *Medicago sativa* is a complex quantitative trait, and neither a single physiological parameter nor an individual gene can adequately explain its ability to withstand low temperatures (Kidokoro et al., 2022; Yao et al., 2023). Currently, the physiological and molecular mechanisms underlying the winter survival of *Medicago sativa* root systems remain unclear. Therefore, this study investigated *Medicago sativa* root system adaptive responses during the overwintering period, aiming to elucidate cold resistance mechanisms through a combined analysis of physiological indicators and cold-responsive gene expression. These findings are anticipated to provide a foundation for molecular breeding of cold-tolerant *Medicago sativa* genotypes suitable for high-altitude regions predominantly characterized by freezing conditions during the winter.

## 2 Materials and methods

### 2.1 Experimental design

The cold-tolerant *Medicago sativa* variety Longmu801 and the cold-sensitive variety Sardi were selected as experimental materials. Longmu801, classified as a fall dormancy level 1 variety, was provided by the Heilongjiang Academy of Agricultural Sciences. The Sardi variety, with a fall dormancy level of 7, was supplied by Bailv (Tianjin) International Grass Industry Co., Ltd. A total of 100 plump *Medicago sativa* seeds were selected and placed in Petri dishes (diameter: 110 mm) lined with double-layer filter paper. Initially, 10 mL of distilled water was added to each plate. Subsequently, the culture dishes were transferred into an illuminated incubator, maintained at a temperature of 25°C, with an 80% humidity level and a light intensity of 15,000 lux. Light and dark cycles were alternated every 12 hours. Following a three-day incubation period for germination, ten seedlings were selected and transplanted into experimental pots. Each pot, measuring 29 cm in height and 25 cm in diameter, was filled with 10 kg of soil collected from the experimental field. The soil was sieved through a 4 mm mesh to remove stones and weed roots. The major soil properties were as follows: organic matter of 29.41 g·kg^-1^, total nitrogen at 1.73 g·kg^-1^, available phosphorus measured at 42.28 mg·kg^-1^, available potassium at 249.45 mg·kg^-1^, and a pH value of 8.3. The experiment commenced on May 10, with irrigation applied every three days. Thinning was carried out when seedlings developed 5–6 leaves to retain eight plants per pot. Soil moisture was maintained at 75%. Subsequently, *Medicago sativa* seedlings were grown under regular field conditions. The experimental site was located in Qiqihar (E 123°24′, N 47°9′), Heilongjiang, China, where the minimum winter temperature reached -34.3°C, and the accumulated temperature (≥10°C) was 1470°C during the year when the experiments were conducted. On September 20, *Medicago sativa* plants from the control group (CK) were transferred to an intelligent greenhouse and were grown under controlled conditions: temperature, 20°C/16°C (light/dark); light and dark cycles were alternated every 12 hours; light intensity of 20,000 lux; and 75% soil moisture. The control group consisted of (A) CK_Longmu801 and (B) CK_Sardi. The experimental treatment group (YD) consisted of *Medicago sativa* grown under natural overwintering conditions: (C) YD_Longmu801 and (D) YD_Sardi. Each treatment was replicated three times. During the overwintering period, Longmu801 and Sardi root samples from both the treatment (YD) and control (CK) groups were collected concurrently on December 25. The roots were washed with distilled water. Some root samples were tested for physiological indices. Physiological indices, including POD (Polle et al., 1994), SS (Buysse and Merckx, 1993), Pro (Bates et al., 1973), GSH (Griffith, 1980), malondialdehyde (MDA) (Guo et al., 2007), ABA (Castonguay et al., 1993), and JA (Šimura et al., 2018) contents were measured. The remaining portion of the root sample was frozen in liquid nitrogen at -80°C for RNA-Seq.

### 2.2 RNA-Seq library construction

Total RNA was first extracted from *Medicago sativa* roots using an RNA extraction kit (Thermo Fisher Scientific Inc., USA), with three biological replicates for each treatment. The concentration of RNA was assessed using a Qubit 3.0 fluorometer (Life Technologies Inc., USA). The RNA integrity number (RIN) was measured using an Agilent 2100 BIOanalyzer (Agilent Technologies Inc., USA). The mRNA was then enriched from 50 μL of total RNA utilizing the Next Oligo (dT) 25 kit (New England Biolabs Inc., USA). The isolated mRNA was reverse-transcribed and used to construct cDNA library with the NEBNext UltraExpress™ kit (Illumina Inc., USA) for subsequent transcriptome sequencing.

### 2.3 Illumina sequencing

Total RNA extracted from *Medicago sativa* roots was sequenced utilizing the Illumina HiSeq platform (Shanghai Majorbio Technology Co., Ltd., China), and generating paired-end reads of 2 × 150 bp. After quality control of the raw sequencing data, low-quality reads and excessively short sequences were removed to obtain high-quality clean data, ensuring the accuracy of subsequent analyses. The RNA-seq data can be found in the NCBI database under accession number PRJNA1221592.

### 2.4 DEG analysis and annotation

The processed RNA-seq data from *Medicago sativa* were assembled *de novo* utilizing Trinity software in combination with the reference genome of *Medicago sativa* cv. Zhongmu No.1 (He et al., 2022). According to the reference genome alignment results, differential splicing and fusion gene detection analyses were conducted. The DEGs were screened for further analysis. The DEGs were annotated by aligning sequences to public databases, including the NCBI Nr database (https://www.ncbi.nlm.nih.gov/public/), the GO database (http://www.geneontology.org), and the KEGG database (http://www.genome.Jp/kegg/) using the Blastx algorithm with an E-value threshold of less than 1 × 10^-5^. The DEG functional annotations were obtained based on accurate sequence alignments. The DEG identification was performed utilizing the DESeq R package. Genes with a *P*-value ≤ 0.05 and an absolute |log2 fold change| > 1 were considered significantly differentially expressed. Subsequently, these DEGs were subjected to GO and KEGG enrichment analyses to explore their functional significance.

### 2.5 Statistical analysis

Statistical analyses were conducted utilizing SAS version 9.0 (SAS Institute Inc., USA) employing ANOVA to identify statistically significant differences among treatments and genotypes. The dataset from *Medicago sativa* roots across the four treatment groups fulfilled normal distribution T-test assumptions. The statistical significance of the differences among the primary treatment groups was evaluated using Duncan’s multiple range T-test, with *P*-values below 0.05 considered significant.

## 3 Results

### 3.1 Physiological characteristics

**Figure 1:**
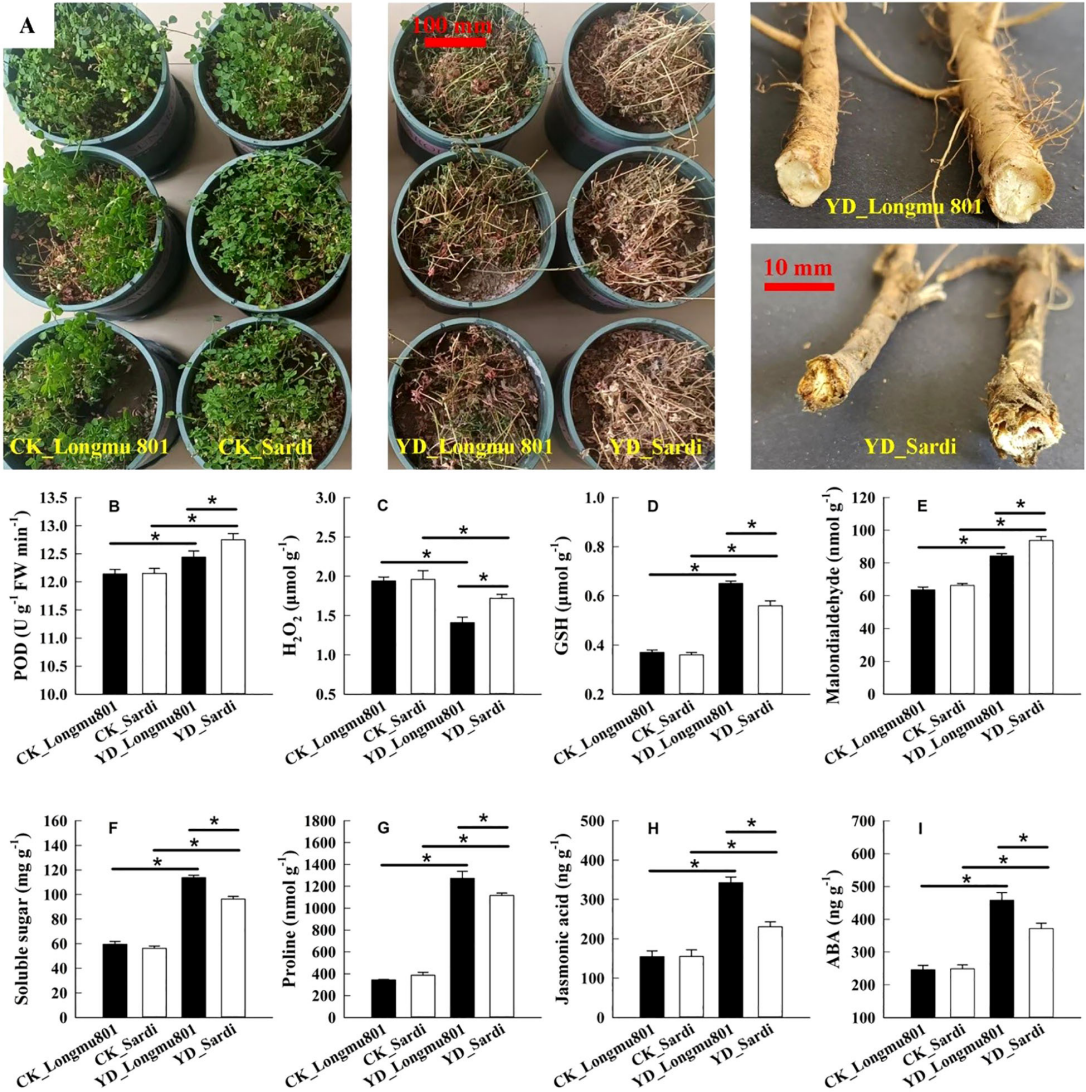
Comparative evaluation of physiological characteristics between *Medicago sativa* varieties Longmu801 (cold-tolerant) and Sardi (cold-sensitive). Root morphology during winter **(A)**, POD activity **(B)**, H_2_O_2_ content **(C)**, GSH content **(D)**, MDA content **(E)**, soluble sugar content **(F)**, proline content **(G)**, JA content **(H)**, and ABA content **(I)**. The asterisk (*) indicates a statistically significant difference between the two varieties (*P* < 0.05).

During the wintering period, the roots of YD_Longmu801 *Medicago sativa* displayed superior vitality compared to YD_Sardi (**Figure 1A**). The POD, hydrogen peroxide (H_2_O_2_), and MDA were significantly higher in the YD_Sardi treatment compared to YD_Longmu801 (**Figures 1B, C, E**), exhibiting a 1.02-fold, 1.22-fold, and 1.11-fold increase, respectively. The root GSH, soluble sugar, and proline contents in the YD_Longmu801 treatment were 1.16-fold, 1.18-fold, and 1.14-fold greater compared to YD_Sardi (**Figures 1D, F, G**). JA and ABA contents in *Medicago sativa* were significantly higher in YD_Longmu801 roots compared to YD_Sardi, with *P*-values below 0.05 (**Figures 1H, I**).

### 3.2 KEGG and GO analysis

**Figure 2:**
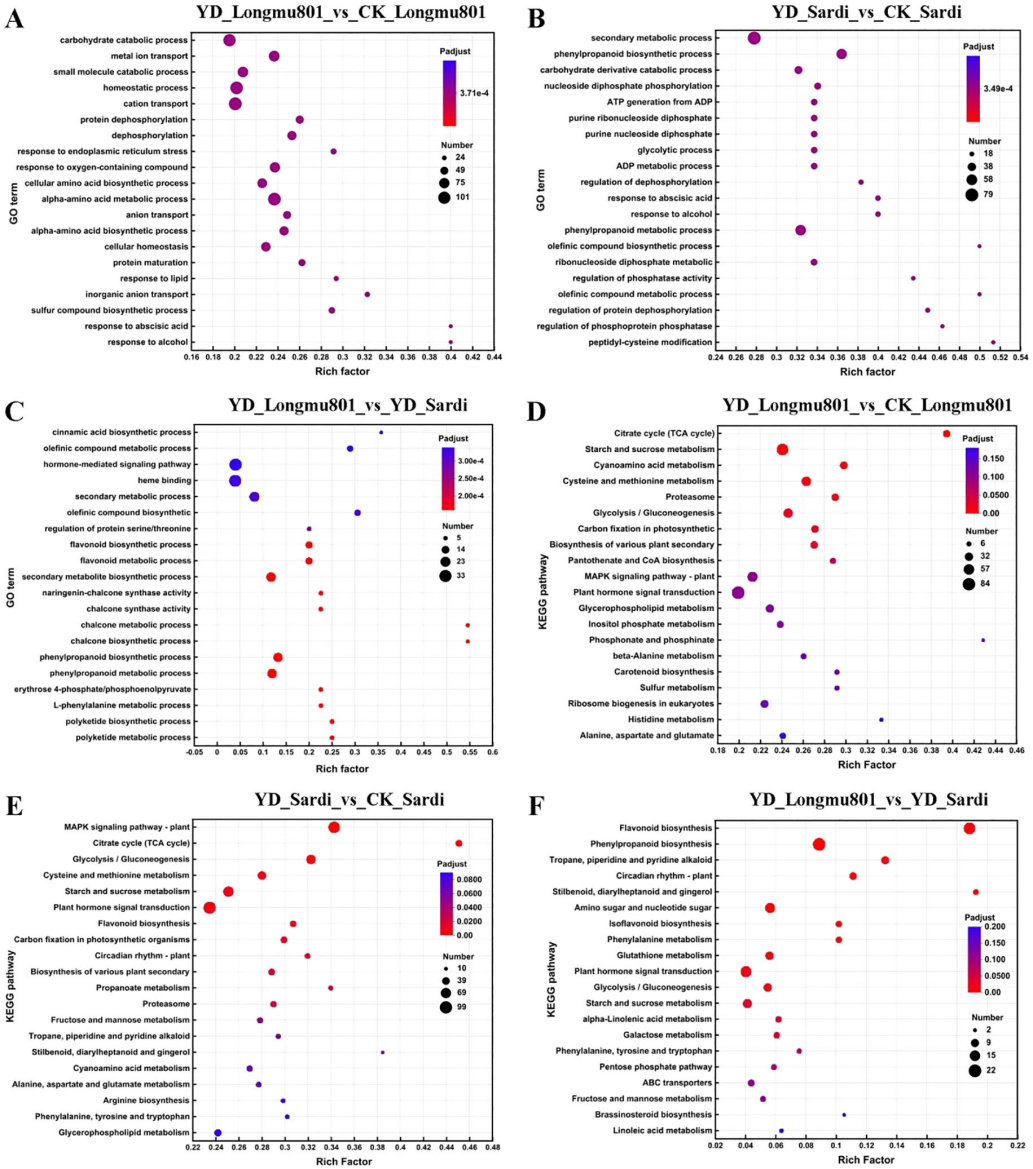
Comparative KEGG and GO analyses between the two *Medicago sativa* varieties under CK and low temperature conditions. GO analysis of DEGs in YD_Longmu801_vs_CK_Longmu801 **(A)**, YD_Sardi_vs_CK_Sardi **(B)**, and YD_Longmu801_vs_YD_Sardi **(C)**. KEGG analysis of DEGs in YD_Longmu801_vs_CK_Longmu801 **(D)**, YD_Sardi_vs_CK_Sardi **(E)**, and YD_Longmu801_vs_YD_Sardi **(F)**. The DEGs were selected using a threshold of *P*-value < 0.05. The size of the dots in the figure represents the number of DEGs, and the color of the dots corresponds to different P_adjust_ ranges.

A total of 113,239 unigenes were obtained from the *Medicago sativa* root transcriptome sequencing (**Supplementary Figure S1A**). A notable positive association was identified between YD_Longmu801 and YD_Sardi, exhibiting a correlation coefficient of 0.940 (**Supplementary Figure S1B**). A total of 34,237 unigenes were annotated, accounting for 30.23% of the total unigenes. The unigenes annotated in the Nr, GO, and KEGG databases were 34,048, 30,405, and 12,807, respectively (**Supplementary Figure S1C**). In the GO analysis, the DEGs in the YD_Longmu801_vs_CK_Longmu801 comparison were mainly enriched in the “carbohydrate catabolic process” and “homeostatic process” categories (**Figure 2A**). The DEGs in the YD_Sardi_vs_CK_Sardi comparison were mainly enriched in the “secondary metabolic” and “phenylpropanoid biosynthetic” categories (**Figure 2B**). In YD_Longmu801_vs_YD_Sardi, DEGs were primarily enriched in the “hormone-mediated signaling pathway” and “heme binding” GO categories (**Figure 2C**). Regarding KEGG enrichment analyses, the DEGs in YD_Longmu801_vs_CK_Longmu801 were predominantly enriched in the “starch and sucrose metabolism” and “plant hormone signal transduction” pathways (**Figure 2D**). DEGs in YD_Sardi_vs_CK_Sardi were mainly enriched in the “MAPK signaling pathway-plant” and “plant hormone signal transduction” pathways (**Figure 2E**). On the other hand, DEGs in YD_Longmu801_vs_YD_Sardi were mainly enriched in the “flavonoid biosynthesis” and “phenylpropanoid biosynthesis” pathways (**Figure 2F**).

### 3.3 DEGs functional annotation

**Figure 3:**
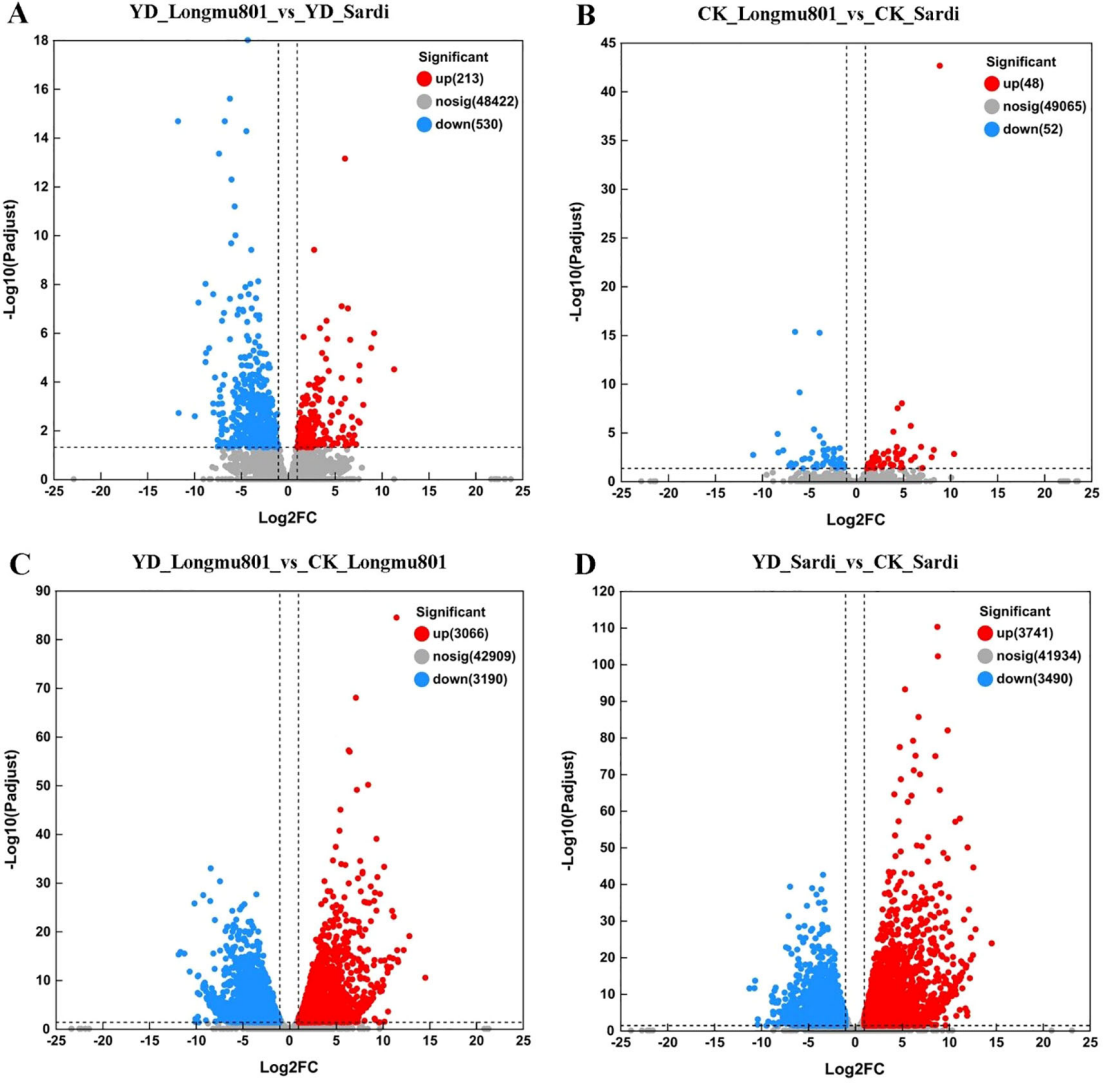
Distinct sets of DEGs characterize the comparison between *Medicago sativa* varieties Longmu801 and Sardi. YD_Longmu801_vs_YD_Sardi **(A)**, CK_Longmu801_vs_CK_Sardi **(B)**, YD_Longmu801_vs_CK_Longmu801 **(C)**, and YD_Sardi_vs_CK_Sardi **(D)**. The DEGs were chosen utilizing a threshold of *P* < 0.05, and |Log2 fold change| > 1. In this analysis, upregulated DEGs are shown in red, while downregulated DEGs are shown in blue.

A total of 14,330 DEGs were identified using the BLAST2GO analysis tool. Among them, 743 DEGs were identified in the YD_Longmu801 vs. YD_Sardi comparison, including 213 upregulated and 530 downregulated DEGs (**Figure 3A**; **Supplementary Table S1**). 100 DEGs were identified in the CK_Longmu801 vs. CK_Sardi comparison, including 48 upregulated and 52 downregulated DEGs (**Figure 3B**). 6,256 DEGs were identified in the YD_Longmu801 vs. CK_Longmu801 comparison, including 3,066 upregulated and 3,190 downregulated DEGs (**Figure 3C**). Similarly, 7,231 DEGs were identified in the YD_Sardi vs. CK_Sardi comparison, including 3,741 upregulated and 3,490 downregulated DEGs (**Figure 3D**).

**Figure 4:**
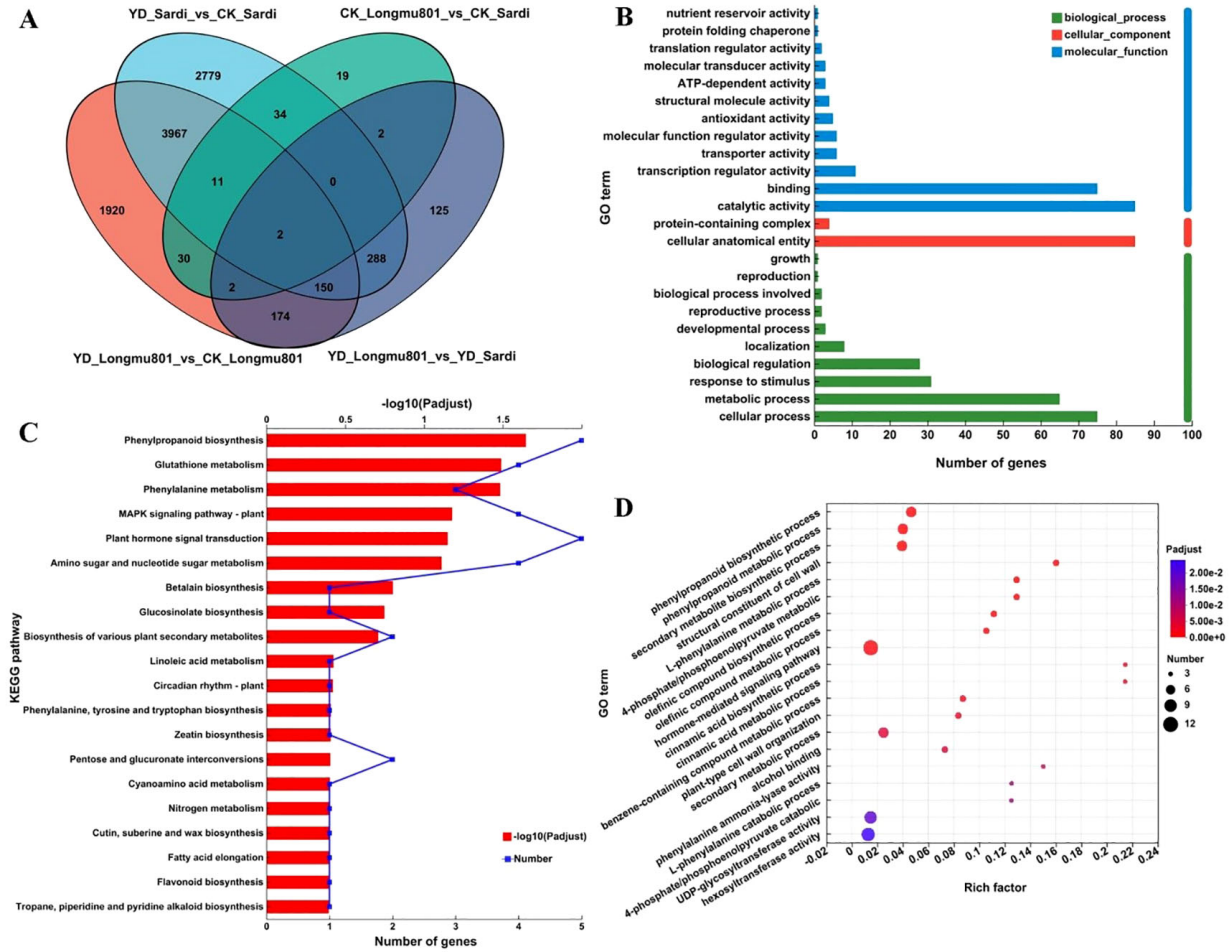
The functional annotation and enrichment analysis of DEGs between *Medicago sativa* varieties. Venn diagram of DEGs **(A)**, GO functional annotation **(B)**, KEGG analysis **(C)**, GO enrichment analysis **(D)**.

As illustrated in the Venn diagram in **Figure 4A**, 1,920, 2,779, and 125 unique DEGs were identified in YD_Longmu801_vs_CK_Longmu801, YD_Sardi_vs_CK_Sardi, and YD_Longmu801_vs_YD_Sardi, with 150 DEGs shared among the treatments. These shared 150 DEGs were functionally annotated and classified using GO terms, and were divided into the three primary GO categories. First, in the biological process category, 85 and 75 DEGs were annotated under “catalytic activity” and “binding”, respectively. Second, in the cellular component category, the highest number of annotated DEGs was found in “cellular anatomical entity”, totaling 85 DEGs. Third, in the molecular function category, DEGs were mainly annotated under “cellular process” (75 DEGs), “metabolic process” (65 DEGs), and “response to stimulus” (31 DEGs) (**Figure 4B**; **Supplementary Table S2**).

Furthermore, the 150 shared DEGs were assigned to 34 KEGG pathways. The KEGG pathways most enriched with DEGs were “phenylpropanoid biosynthesis”, “glutathione metabolism”, “phenylalanine metabolism”, “MAPK signaling pathway”, and “plant hormone signal transduction” (**Figure 4C**; **Supplementary Table S3**). GO enrichment analysis of the DEGs using Goatools assigned the DEGs to 186 GO pathways. The GO pathways most enriched with DEGs were “hormone-mediated signaling pathway”, “secondary metabolite biosynthetic”, and “phenylpropanoid metabolic” (**Figure 4D**; **Supplementary Table S4**).

### 3.4 Analysis of DEGs co-expression network

**Figure 5:**
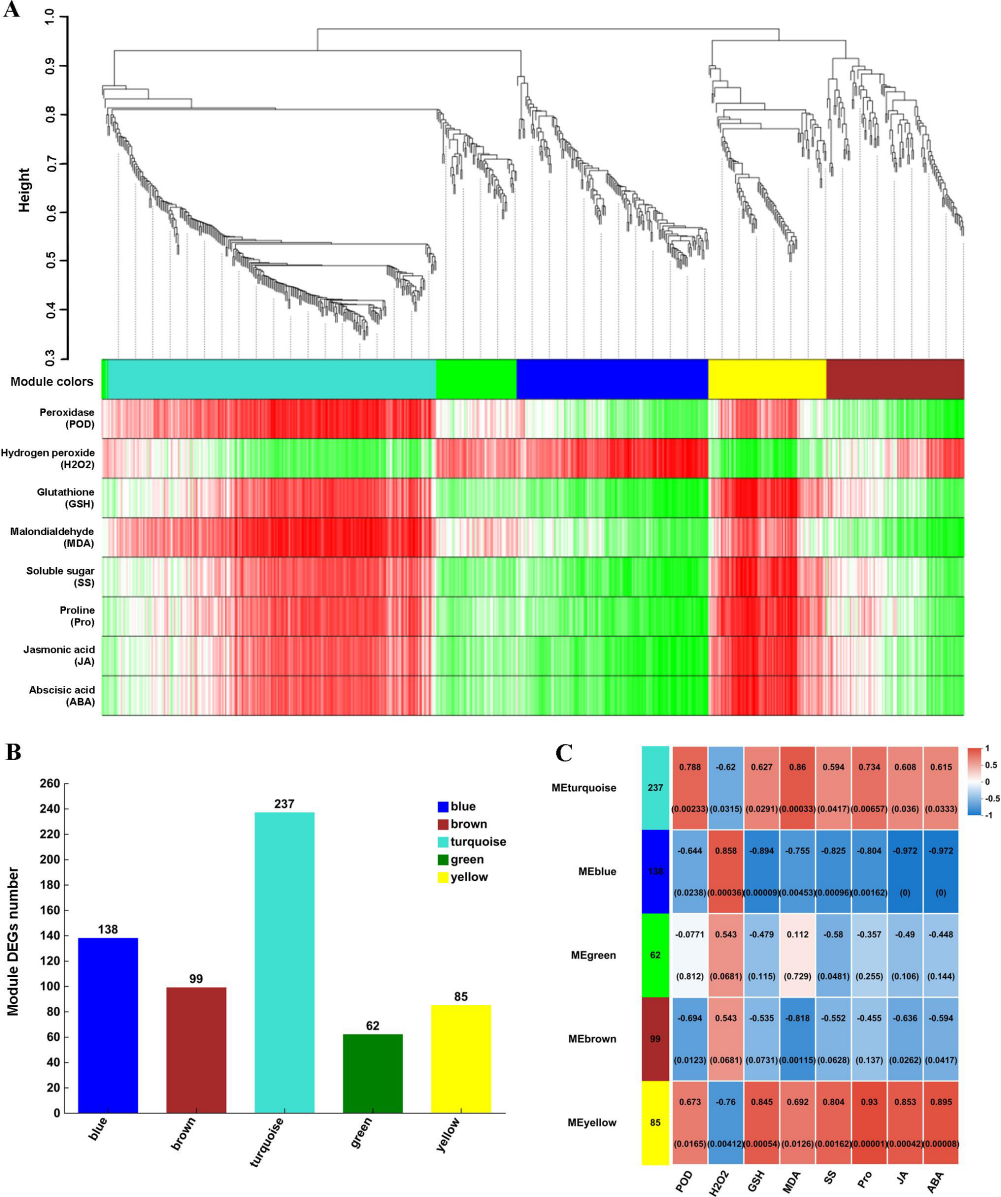
Weighted co-expression network analysis of the DEGs. Hierarchical clustering dendrogram, in which each color in the tree represents a module, and each gene belonging to the same module is indicated by the corresponding color. The vertical distance represents the distance between two genes **(A)**, the number of DEGs in each module **(B)**, and correlations between modules and physiological indicators **(C)**.

In YD_Longmu801_vs_YD_Sardi, 743 DEGs were filtered to eliminate genes with low expression (standard deviation ≤ 0.5), resulting in 621 DEGs, which were used to construct the co-expression network model (**Figure 5A**). The DEGs were divided into five modules: turquoise, blue, brown, yellow, and green, which contained 237, 138, 99, 85, and 62 DEGs, respectively (**Figure 5B**). The correlation analysis between DEG modules and physiological indicators, as illustrated in **Figure 5C**, revealed that the DEGs in the yellow module exhibited a significant positive correlation with POD, GSH, Pro, JA, and ABA (*P* < 0.05), with correlation coefficients of 0.673, 0.845, 0.930, 0.853, and 0.895, respectively. The DEGs in the blue module were significantly negatively correlated with POD, GSH, Pro, JA, and ABA (*P* < 0.05). The turquoise module also exhibited a positive correlation with POD, GSH, Pro, JA, and ABA (*P* < 0.05).

**Figure 6:**
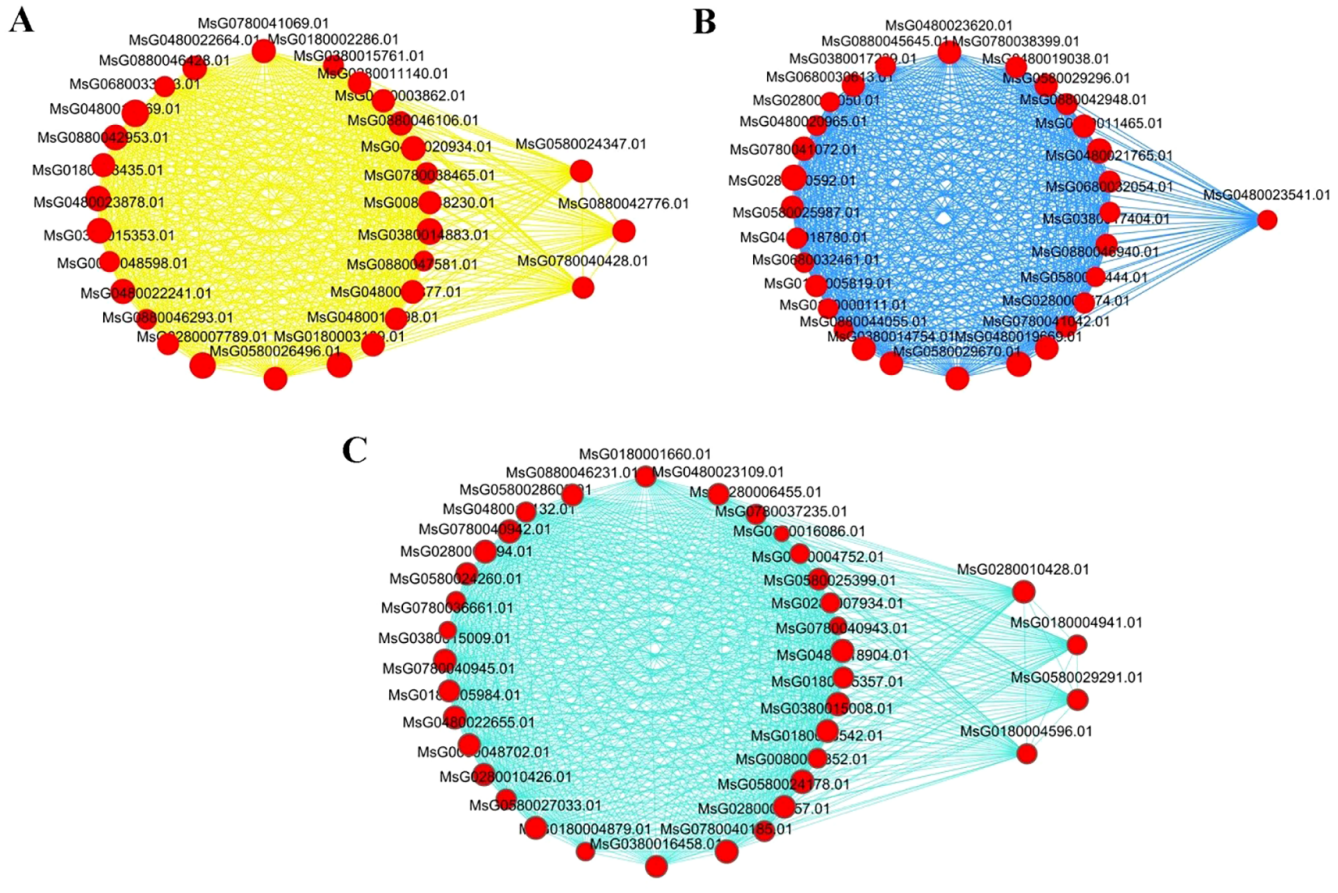
Co-expression networks of selected DEGs within the yellow **(A)**, blue **(B)**, and turquoise **(C)** gene modules. Red nodes outside the circles represent hub genes, while circular nodes within the network represent other genes with relatively high connection strength.

A gene co-expression network was constructed based on the connection strength among the DEGs of the three modules most significantly associated with low temperature stress, namely, the yellow, blue, and turquoise modules (**Figures 6A–C**). The hub genes with the highest connection strength were selected as key genes associated with cold tolerance in *Medicago sativa*. These included one gene from the blue module, three genes from the yellow module, and four genes from the turquoise module.

**Figure 7:**
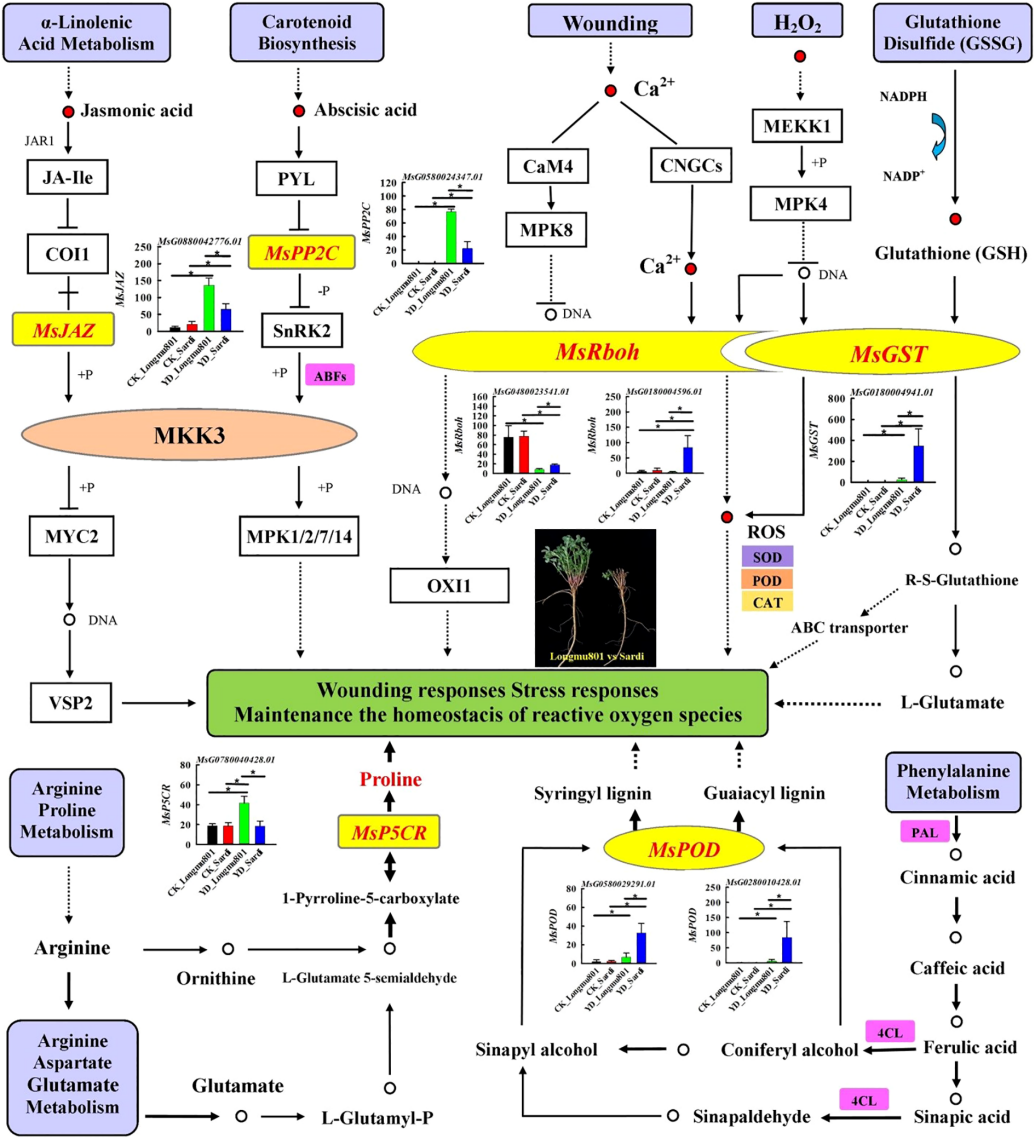
Pathways related to the regulation of DEGs in areas, including “plant hormone signal transduction”, “glutathione metabolism”, “MAPK signaling pathway”, “arginine and proline metabolism”, and “phenylpropanoid biosynthesis”.

The hub genes in the yellow, blue, and turquoise modules mainly participate in regulatory pathways such as “plant hormone signal transduction”, “glutathione metabolism”, “MAPK signaling pathway”, “arginine and proline metabolism”, and “phenylpropanoid biosynthesis”. Specifically, in the pathways of “plant hormone signal transduction”, *MsPP2C* (protein phosphatase 2C; *MsG0580024347.01*) and *MsJAZ* (jasmonate ZIM domain protein; *MsG0880042776.01*) are involved in the biosynthesis and signaling of ABA and JA. The expression levels of *MsPP2C* and *MsJAZ* in YD_Longmu801 were significantly higher than those in CK_Longmu801 and YD_Sardi (*P* < 0.05). In the “MAPK signaling pathway”, and “glutathione metabolism”, DEGs related to ROS (H_2_O_2_) pathway in plants, such as *MsRboh* (respiratory burst oxidase homologues; *MsG0480023541.01*, *MsG0180004596.01*) and *MsGST* (glutathione-S-transferase; *MsG0180004941.01*), were downregulated. In the pathways of “arginine and proline metabolism” and “phenylpropanoid biosynthesis”, *MsP5CR* (pyrroline-5-carboxylate reductase; *MsG0780040428.01*) and *MsPOD* (peroxidase; *MsG0280010428.01, MsG0580029291.01*) involved in the biosynthesis of proline and phenylalanine (**Figure 7**).

## 4 Discussion

Low temperature is a significant abiotic stress factor impacting the growth, yield, and quality of *Medicago sativa*, grown in the high-latitude areas of northern China, frequently encounters extreme low temperatures in winter, which increase root vulnerability to frost damage, subsequently resulting in a decrease in both its quality and yield (Liu et al., 2019). This study aimed to explore the biological and molecular regulatory processes in *Medicago sativa* in response to low-temperature stress. Using RNA-seq transcriptome analyses, key DEGs were identified in the roots in the YD_Longmu801 vs. YD_Sardi comparison: *MsGST*, *MsRboh*, *MsPOD*, *MsP5CR, MsPP2C*, and *MsJAZ*. Furthermore, these DEGs demonstrated significant positive correlations with GSH, POD, ABA, proline, and JA levels. The DEGs are mainly related to pathways such as “glutathione metabolism”, “plant hormone signal transduction”, “MAPK signaling pathway”, “arginine and proline metabolism”, and “phenylpropanoid metabolism” (Shu et al., 2017).

GSTs in plants are involved in responses to pathogen invasion, osmotic regulation, and low-temperature stress. Zhao et al. (2015) revealed that low-temperature stress resulted in an increased expression of *GST* genes in cold-tolerant rice varieties. These findings are consistent with the results reported above. Under low temperatures, the expression levels of *MsGST* and the GSH content in the roots of YD_Longmu801 and YD_Sardi were higher than the CK (**Figure 1D**). Moreover, their H_2_O_2_ content was lower compared the CK (**Figure 1C**), indicating that *MsGST*, through the conjugation of reduced glutathione with endogenous/exogenous electrophilic groups, catalyzes the formation of conjugates such as R-S-glutathione, which can transferred via ATP-binding cassette (ABC) transporters across membranes, thereby protecting plant cells from ROS (e.g., H_2_O_2_) damage (Wang et al., 2024b; Song et al., 2019). Moreover, significant differences in *MsGST* gene expression were observed between the two *Medicago sativa* varieties during the winter. The expression levels of *MsGST* in YD_Sardi were significantly higher than in YD_Longmu801 (**Figure 7**), while GSH content was significantly lower in YD_Sardi (**Figure 1D**). This suggests that the overexpression of *MsGST* genes during the overwintering process reduced GSH content, while increasing H_2_O_2_ and MDA contents (**Figure 1E**). H_2_O_2_ accumulation, resulting from the overexpression of *MsGST* and the concomitant reduction of GSH, potentially activates the biosynthesis pathways of glutamic acid and gamma-glutamylcysteine, thereby inhibiting root growth through the formation of glutamate during the process of glutathione-mediated reactive oxygen species (ROS) detoxification (Walch-Liu et al., 2006). The accumulation of MDA in the cold-sensitive Sardi *Medicago sativa* was a result of more severe membrane lipid peroxidation, indicating that during excessive ROS accumulation (oxidative burst), GST and GPX (glutathione peroxidases) proteins are significantly induced to aid the detoxification of lipid peroxides, DNA degradation products, and ROS. Further, increased cellular levels of ROS, which are second messengers during cold stress conditions, lead to PCD (programmed cell death) in cold-sensitive *Medicago sativa* (Song et al., 2019; Vijayakumar et al., 2016). However, the lower contents of H_2_O_2_ and MDA in Longmu801 indicated less damage to root cell membranes, contributing to its stronger cold tolerance.

Rboh belongs to the NADPH oxidase family. The roots of YD_Longmu801 *Medicago sativa* exhibited a marked decrease in the expression level of *MsRboh* during the wintering period (**Figure 7**), and the patterns of H_2_O_2_ content changes were similar to that of *MsRboh* gene expression (**Figure 1C**). This suggests that the oxidative stress response in Longmu801 roots during winter is generally well-controlled, which explains the low expression of the *MsRboh* and the reduced accumulation of ROS such as H_2_O_2_. However, in the “MAPK signaling pathway”, the expression level of *MsRboh* (*MsG0480023541.01*, *MsG0180004596.01*) in YD_Sardi was significantly higher than in YD_Longmu801, aligning with the findings of Zhou et al. (2012). This indicates that the *MsRboh* gene in the roots of cold-sensitive Sardi *Medicago sativa* remains relatively upregulated during the overwintering period. It has been established that Rboh catalyzes the production of superoxide anion (O_2_·-) from extracellular O_2_ by using NADPH in the root cytoplasm as an electron donor while also generating H_2_O_2_ (Devireddy et al., 2021). In this process, Ca^2+^ regulates the activity of CaM_4_ and CNGC channel proteins through indirect phosphorylation, leading to the overexpression of *MsRboh* genes, which in turn activate SOD and POD enzymes to degrade ROS, including H_2_O_2_ and O_2_·- (Devireddy et al., 2021; Song et al., 2014). ROS can be produced through multiple pathways such as glutathione metabolism and MAPK signaling pathway. Excessive accumulation of H_2_O_2_ in root cells under low temperatures can damage cell membrane structures and cellular components, ultimately resulting in PCD and significant damage to the roots of the Sardi *Medicago sativa* variety (Suzuki and Mittler, 2010). During the overwintering period, the POD activity in the root system of YD_Sardi was significantly higher compared to YD_Longmu801 (**Figure 1B**). Furthermore, in the pathway of “phenylpropanoid biosynthesis”, RNA-seq data also confirmed that the expression levels of *MsPOD* (*MsG0280010428.01*, *MsG0580029291.01*) genes in YD_Sardi were higher than in YD_Longmu801, which is consistent with the findings reported by Yang et al. (2023). This suggests that the cold-sensitive Sardi variety exhibits increased POD activity and biosynthesis of guaiacyl and syringyl lignin through the upregulation of *MsPOD*. This upregulation eliminates excessive peroxides and induces ferulic acid and lignin accumulation in the phloem of roots (Baek and Skinner, 2003; Yang et al., 2023). Consequently, it protects cold-sensitive *Medicago sativa* roots from oxidative stress damage. Recent studies have characterized *POD*, *PAL* (phenylalanine ammonia-lyase), and *4CL* (4-coumarate-CoA ligase) genes as playing crucial roles in the biosynthetic pathways of guaiacyl and syringyl lignin in the abiotic stress (Wang et al., 2023; Yang et al., 2023). On the other hand, the cold-tolerant Longmu801 variety accumulates lower levels of O_2_·- and H_2_O_2_ in its roots, enabling more effective activation of cell membrane regulation and antioxidant signaling pathways in response to cold.

The phytohormones ABA and jasmonic acid are essential in the regulation of plants’ responses and tolerance to cold stress conditions (Kazan, 2015). Under low-temperature stress, the ABA content in the roots of the cold-tolerant variety Longmu801 was significantly increased. ABA regulates cellular metabolism by modulating the activity of protein phosphatase 2C (PP2C) (Santner and Estelle, 2009), a key component in the ABA signaling pathway. PP2C regulates the kinase activity of SnRK2 or MAPK, thereby enhancing cold tolerance in various plant species, such as *Medicago truncatula* (Yang et al., 2018), *Nicotiana tabacum* (Hu et al., 2010), and *Arabidopsis thaliana* (Tähtiharju and Palva, 2001). In this study, the expression of *MsPP2C* in YD_Longmu801 was significantly higher compared to YD_Sardi, indicating that *MsPP2C* is a transcription factor with crucial signaling functions in *Medicago sativa* responses to low-temperature stress. This discrepancy is probably attributable to the inactivation of *PP2C* by ABA-binding receptor proteins of the PYR1-like (PYLs) type, which leads to the dephosphorylation of SnRK2 kinase by PP2C and its subsequent activation (Fujii et al., 2009). This culminates in the establishment of the ABA-PYL-PP2C-SnRK2 signaling pathway, whose activation leads to the phosphorylation of bZIP (basic leucine zipper) trans-acting factors known as *ABFs* (ABA-associated factors) and *ABREs* (ABA-responsive promoter elements), which in turn induce the transcription of *CBFs/DREBs* genes, enhancing *Medicago sativa* cold tolerance (Wei et al., 2015). Furthermore, JA is intricately associated with the cold tolerance of *Medicago sativa*. Increased JA content positively modulates the ICE-CBF-COR transcriptional pathway, thereby enhancing plant cold resistance (Feng et al., 2025; Hu et al., 2017). A similar pattern was observed in this study. During winter, the JA content in the roots of YD_Longmu801 *Medicago sativa* was higher than in YD_Sardi, while the expression of *MsJAZ* in the roots of YD_Longmu801 was significantly upregulated. *MsJAZ* activates MKK3 (MAP Kinase Kinase 3), leading to the dissociation of the transcription factor *MYC2* and the regulation of DNA transcription in response to low-temperature stress. This suggests that the cold-resistant variety Longmu801 exhibits increased expression of the *MsJAZ* gene through the ICE-CBF regulatory pathway, which in turn releases the *CBF* transcription factors. *CBF* binds to the promoter of the *COR* genes and induces *COR* gene expression, thereby enhancing *Medicago sativa* cold resistance (Feng et al., 2025). Therefore, it may be concluded that during winter, the *MsPP2C* and *MsJAZ* genes in the *Medicago sativa* roots positively regulate the CBF-COR signaling pathway, resulting in elevated levels of the endogenous plant hormones ABA and JA, which in turn enhance *Medicago sativa*’s cold tolerance (Shu et al., 2017).

Proline can accumulate in plants under stress conditions such as low temperature, drought, and salinity (Repkina et al., 2021). Proline is involved in the regulation of cell osmotic potential and helps maintain turgor pressure in cells under stress. It also aids plants in enduring dehydration, with minimal or no detrimental effects (Zhuo et al., 2018). Moreover, it has been demonstrated that proline content increases under low-temperature conditions (Wang et al., 2024b). The buildup of proline under stress conditions might occur due to increased production or decreased breakdown of proline. Cold-tolerant *Medicago sativa* Longmu801 showed a notable increase in proline content under low temperature stress, in contrast to Sardi *Medicago sativa* (**Figure 1G**). In addition, the KEGG pathway “arginine and proline metabolism” demonstrated a significant increase in enrichment. Proline is synthesized from glutamate and arginine in a process catalyzed by two enzymes, P5CS (pyrroline-5-carboxylate synthase) and P5CR (pyrroline-5-carboxylate reductase) (Repkina et al., 2021). Among the DEGs, *MsP5CR* was significantly more highly expressed in *Medicago sativa* YD_Longmu801 compared to YD_Sardi. This indicated that the biosynthetic pathway of proline was further activated by *MsP5CR*, in addition to ornithine conversion to P5C (1-pyrroline-5-carboxylate) by the catalytic action of *OAT* (ornithine aminotransferase) (Wei et al., 2022). Through the cold stress-induced upregulation of *MsP5CR*, proline was produced by P5C, leading to the accumulation of proline in Longmu801 *Medicago sativa*, which in turn enhanced the root’s cold tolerance.

Weighted gene co-expression network analysis (WGCNA) has been extensively implemented in stress resistance research, as it is currently the most efficient method to identify correlations among genes (Ovens et al., 2021). Furthermore, WGCNA assists in characterizing the molecular functions of gene modules comprising co-expressed genes (Yin et al., 2018). The approach involves conducting correlation analysis between co-expressed genes and plant physiological characteristics, to effectively identify hub genes associated with these traits. Cui et al. (2019) applied WGCNA to study low-temperature-responsive hub genes in *Medicago falcata*, identifying key transcription factors and genes within relevant modules. Similarly, Wang et al. (2022) used WGCNA in their research on freezing-tolerance in *Medicago sativa*, discovering that the transcription factors *ABCC8* and *ABCC3* may serve as key downstream regulators of freezing resistance. In this study, we conducted WGCNA using physiological indicators and RNA-seq data, identifying that *MsGST*, *MsRboh*, *MsPOD*, *MsJAZ*, *MsP5CR*, and *MsPP2C* as potential key regulators of low-temperature tolerance in *Medicago sativa*. The WGCNA between DEGs and physiological indices revealed that DEGs in the yellow, blue, and turquoise modules were significantly correlated with POD, GSH, proline, JA, and ABA levels. The hub genes from the three cold tolerance-associated modules were significantly enriched in numerous major metabolic pathways involved in stress tolerance, including plant hormone signal transduction, glutathione metabolism, the MAPK signaling pathway, arginine and proline metabolism, and phenylpropanoid metabolism. Many previous studies have investigated the effects of amino acids, plant hormones, and glutathione on the cold tolerance of *Medicago sativa* (Shu et al., 2017; Xu et al., 2024; Zhang et al., 2024). Through WGCNA and correlation analysis, this study identified hub genes and explored their potential relationships with physiological characteristics. This deepens our understanding of these genes and provides a solid theoretical foundation for future research on cold tolerance mechanisms in *Medicago sativa*.

## 5 Conclusion

During the winter period, a total of 743 DEGs were identified in the roots of cold-tolerant and cold-sensitive *Medicago sativa* varieties. Changes in jasmonic acid, ABA, POD, proline, and glutathione levels were consistent with the expression differences of DEGs associated with cold resistance. Finally, key DEGs involved in low-temperature stress signal transduction pathways were identified, including *MsGST*, *MsRboh*, *MsPOD*, *MsJAZ*, *MsP5CR*, and *MsPP2C*. The findings of this study provide genetic resources for the research of functional genomics of *Medicago sativa* under low-temperature stress and provide a theoretical reference and target candidate genes for the molecular breeding of winter hardiness in *Medicago sativa* cultivated in cold regions.

## Data Availability

The transcriptome data were saved in the NCBI database PRJNA1221592, and SRA with accession numbers SRR32297686, SRR32297683, SRR32297682, SRR32297687, SRR32297684, SRR32297685, SRR32297680, SRR32297679, SRR32297678, SRR32297676, SRR32297677, and SRR32297681.
