# Supplementary figures and images for: Analysis of physiological characteristics and gene co-expression networks in Medicago sativa roots under low-temperature stress
Source: Front Plant Sci. 2025 Aug 25;16:1597949. doi: 10.3389/fpls.2025.1597949 (PMC12415042; doi:10.3389/fpls.2025.1597949)

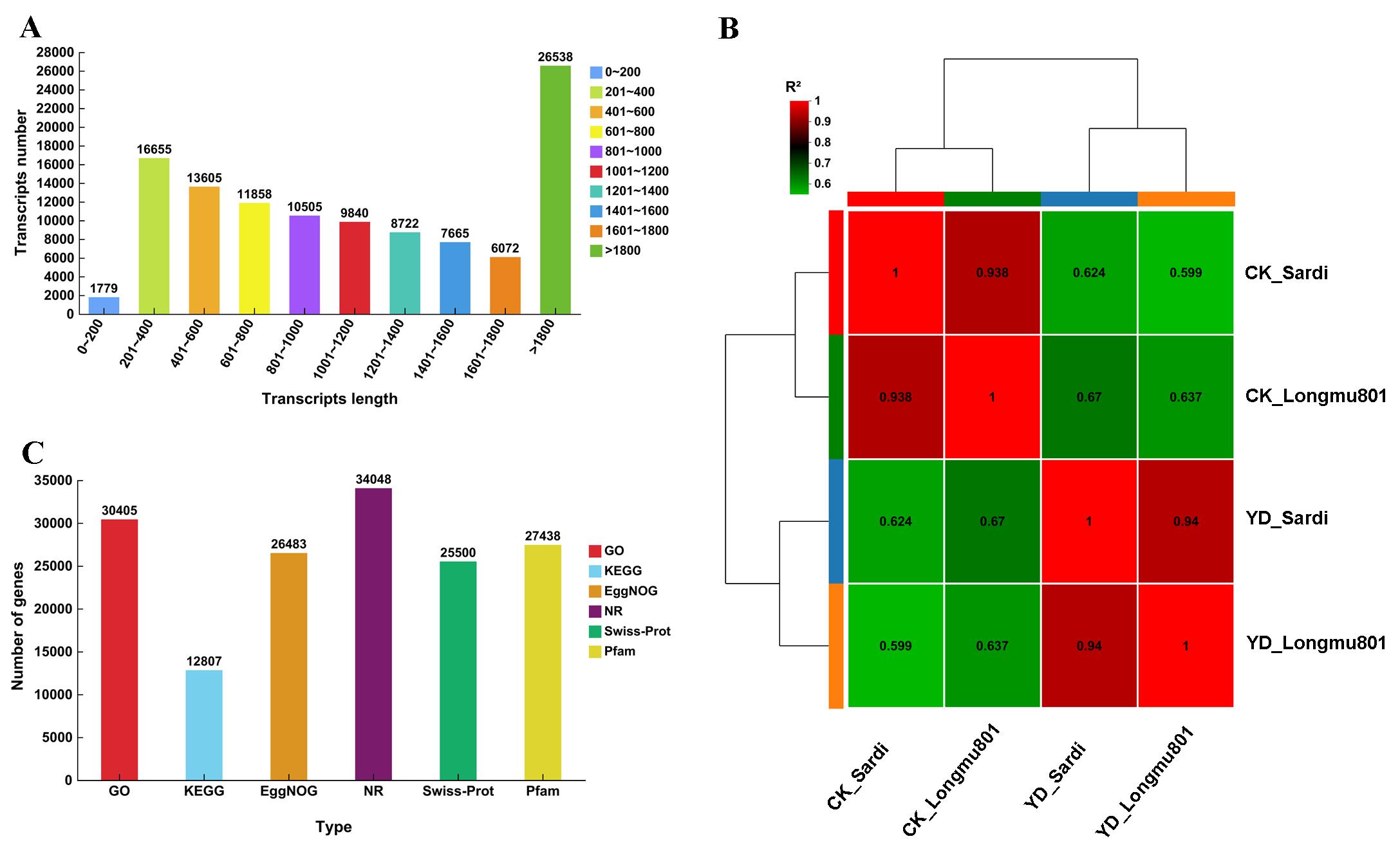

Supplement: Supplementary file 1 [file Image1.jpeg]
