# Supplementary material for: Analysis of physiological characteristics and gene co-expression networks in Medicago sativa roots under low-temperature stress
Source: Front Plant Sci. 2025 Aug 25;16:1597949. doi: 10.3389/fpls.2025.1597949 (PMC12415042; doi:10.3389/fpls.2025.1597949)
Supplement: Supplementary file 2 [file Table1.docx]

**Differential expression genes in the root of two alfalfa varieties**

| **Gene_id** | **Log_2_FC**  **(YD_Longmu801_vs_YD_Sardi)** | **P-value** | **Significant** | **Regulate** |
| --- | --- | --- | --- | --- |
| MsG0080047831.01 | 4.551591471 | 5.11E-06 | yes | up |
| MsG0080047924.01 | 2.486027482 | 0.001067 | yes | up |
| MsG0080047948.01 | 2.309885412 | 0.000124 | yes | up |
| MsG0080047978.01 | -4.911839232 | 3.24E-05 | yes | down |
| MsG0080048036.01 | -5.176968529 | 0.00151 | yes | down |
| MsG0080048147.01 | -4.685307174 | 0.000136 | yes | down |
| MsG0080048230.01 | 2.799872024 | 2.44E-13 | yes | up |
| MsG0080048346.01 | -4.751366937 | 1.87E-10 | yes | down |
| MsG0080048348.01 | -5.78375704 | 4.42E-05 | yes | down |
| MsG0080048352.01 | -4.811481901 | 1.57E-10 | yes | down |
| MsG0080048387.01 | -6.730394438 | 3.65E-19 | yes | down |
| MsG0080048597.01 | -1.442258628 | 8.89E-05 | yes | down |
| MsG0080048598.01 | 1.410759572 | 0.000864 | yes | up |
| MsG0080048609.01 | 1.750716608 | 0.000622 | yes | up |
| MsG0080048702.01 | -1.723951129 | 5.82E-05 | yes | down |
| MsG0080048716.01 | -2.096358845 | 0.000924 | yes | down |
| MsG0080048939.01 | 2.410887855 | 1.76E-05 | yes | up |
| MsG0080049045.01 | -1.91983844 | 0.001477 | yes | down |
| MsG0080049073.01 | 7.619377398 | 4.22E-07 | yes | up |
| MsG0080049149.01 | -4.301060554 | 7.95E-05 | yes | down |
| MsG0180000076.01 | -2.403396159 | 0.000194 | yes | down |
| MsG0180000111.01 | -1.842546037 | 0.001217 | yes | down |
| MsG0180000162.01 | -4.084339354 | 0.000554 | yes | down |
| MsG0180000181.01 | -7.421664705 | 7.47E-06 | yes | down |
| MsG0180000187.01 | -4.361867708 | 0.000511 | yes | down |
| MsG0180000223.01 | 3.357109219 | 0.000638 | yes | up |
| MsG0180000525.01 | -5.286123586 | 0.00018 | yes | down |
| MsG0180000526.01 | -4.566719183 | 1.47E-06 | yes | down |
| MsG0180000762.01 | -1.677572037 | 0.000675 | yes | down |
| MsG0180000867.01 | -1.664916653 | 0.00106 | yes | down |
| MsG0180000876.01 | 2.02619394 | 9.10E-05 | yes | up |
| MsG0180000883.01 | -4.874920384 | 0.000741 | yes | down |
| MsG0180000983.01 | -3.057798242 | 0.000184 | yes | down |
| MsG0180000984.01 | -2.550099155 | 0.000171 | yes | down |
| MsG0180000993.01 | -2.274689053 | 4.26E-05 | yes | down |
| MsG0180001050.01 | -2.342717739 | 0.000147 | yes | down |
| MsG0180001084.01 | -5.282854773 | 1.25E-05 | yes | down |
| MsG0180001147.01 | 1.467403937 | 3.92E-05 | yes | up |
| MsG0180001149.01 | 1.373799411 | 0.000458 | yes | up |
| MsG0180001166.01 | -1.308048365 | 0.001098 | yes | down |
| MsG0180001251.01 | -3.149449655 | 0.000173 | yes | down |
| MsG0180001447.01 | -3.600057345 | 0.000112 | yes | down |
| MsG0180001480.01 | -1.787329865 | 3.56E-06 | yes | down |
| MsG0180001517.01 | 4.172530815 | 0.000466 | yes | up |
| MsG0180001612.01 | -1.252297398 | 0.001623 | yes | down |
| MsG0180001619.01 | -3.092697529 | 0.000164 | yes | down |
| MsG0180001649.01 | -4.278215675 | 4.99E-23 | yes | down |
| MsG0180001652.01 | -3.99324434 | 8.22E-07 | yes | down |
| MsG0180001654.01 | -3.539870545 | 7.27E-05 | yes | down |
| MsG0180001655.01 | -3.158406465 | 5.33E-12 | yes | down |
| MsG0180001656.01 | -3.018922948 | 8.68E-09 | yes | down |
| MsG0180001660.01 | -1.891447701 | 0.001339 | yes | down |
| MsG0180001676.01 | -5.05276223 | 0.000786 | yes | down |
| MsG0180001786.01 | 8.039780349 | 8.24E-06 | yes | up |
| MsG0180002114.01 | -7.067499309 | 0.000313 | yes | down |
| MsG0180002249.01 | -3.614202372 | 5.45E-05 | yes | down |
| MsG0180002286.01 | 1.236417972 | 0.000592 | yes | up |
| MsG0180002421.01 | -2.570744965 | 0.000276 | yes | down |
| MsG0180002472.01 | -3.693137426 | 1.08E-05 | yes | down |
| MsG0180002993.01 | -4.375210181 | 7.35E-08 | yes | down |
| MsG0180003032.01 | -2.048689981 | 6.27E-08 | yes | down |
| MsG0180003033.01 | -2.585333339 | 0.000106 | yes | down |
| MsG0180003181.01 | 1.166843445 | 0.000201 | yes | up |
| MsG0180003189.01 | 1.701727391 | 0.001404 | yes | up |
| MsG0180003237.01 | -3.996284108 | 7.75E-12 | yes | down |
| MsG0180003252.01 | -4.893111594 | 9.26E-05 | yes | down |
| MsG0180003404.01 | -2.278261394 | 0.000179 | yes | down |
| MsG0180003435.01 | 3.483844166 | 4.76E-07 | yes | up |
| MsG0180003462.01 | -3.9446179 | 0.001208 | yes | down |
| MsG0180003484.01 | -3.310934578 | 0.000118 | yes | down |
| MsG0180003542.01 | -4.180999684 | 2.44E-11 | yes | down |
| MsG0180003620.01 | -2.554967721 | 2.04E-05 | yes | down |
| MsG0180003673.01 | 4.724016987 | 0.001305 | yes | up |
| MsG0180003745.01 | -6.030963873 | 0.000968 | yes | down |
| MsG0180003746.01 | -7.280077344 | 1.27E-06 | yes | down |
| MsG0180003862.01 | 1.217082921 | 0.000548 | yes | up |
| MsG0180003906.01 | 2.707741403 | 0.001436 | yes | up |
| MsG0180003996.01 | -1.740407621 | 0.000607 | yes | down |
| MsG0180004053.01 | -3.625957873 | 3.54E-06 | yes | down |
| MsG0180004094.01 | -9.918386374 | 3.03E-05 | yes | down |
| MsG0180004099.01 | -2.072403144 | 0.0006 | yes | down |
| MsG0180004112.01 | -4.482417482 | 0.001015 | yes | down |
| MsG0180004158.01 | -7.243269615 | 0.000808 | yes | down |
| MsG0180004161.01 | 1.274244019 | 1.92E-05 | yes | up |
| MsG0180004203.01 | -1.520439774 | 0.000461 | yes | down |
| MsG0180004509.01 | -2.169796413 | 0.001801 | yes | down |
| MsG0180004515.01 | 1.590570448 | 6.12E-05 | yes | up |
| MsG0180004596.01 | -4.524085297 | 1.11E-11 | yes | down |
| MsG0180004661.01 | -3.206050104 | 0.000421 | yes | down |
| MsG0180004674.01 | -1.676130878 | 0.001071 | yes | down |
| MsG0180004752.01 | -1.380316491 | 0.000937 | yes | down |
| MsG0180004830.01 | -2.798906797 | 0.000363 | yes | down |
| MsG0180004879.01 | -2.994551056 | 0.00115 | yes | down |
| MsG0180004919.01 | -1.568993174 | 0.000194 | yes | down |
| MsG0180004941.01 | -2.069165559 | 0.00163 | yes | down |
| MsG0180005028.01 | -1.821406997 | 0.000178 | yes | down |
| MsG0180005096.01 | -2.561354799 | 1.40E-05 | yes | down |
| MsG0180005130.01 | -3.122826805 | 2.69E-06 | yes | down |
| MsG0180005143.01 | -2.460515988 | 9.93E-05 | yes | down |
| MsG0180005166.01 | 2.645189217 | 0.000519 | yes | up |
| MsG0180005354.01 | -1.456995572 | 0.001181 | yes | down |
| MsG0180005357.01 | -4.182466513 | 4.70E-09 | yes | down |
| MsG0180005358.01 | -6.810713277 | 2.25E-10 | yes | down |
| MsG0180005372.01 | -11.64491165 | 2.05E-05 | yes | down |
| MsG0180005434.01 | -3.856967197 | 2.62E-06 | yes | down |
| MsG0180005450.01 | 3.725551957 | 3.61E-07 | yes | up |
| MsG0180005555.01 | 2.492198454 | 2.48E-05 | yes | up |
| MsG0180005808.01 | 1.275096301 | 0.001067 | yes | up |
| MsG0180005811.01 | -3.485990477 | 0.000178 | yes | down |
| MsG0180005819.01 | -1.417356109 | 0.000672 | yes | down |
| MsG0180005857.01 | -2.145556359 | 2.25E-05 | yes | down |
| MsG0180005984.01 | -4.708254728 | 6.82E-06 | yes | down |
| MsG0180005996.01 | -5.831966893 | 0.001536 | yes | down |
| MsG0180005997.01 | -2.447568198 | 0.000616 | yes | down |
| MsG0180006187.01 | -3.422884323 | 6.44E-05 | yes | down |
| MsG0180006190.01 | -2.989562324 | 0.000215 | yes | down |
| MsG0280006412.01 | -4.315890568 | 6.20E-05 | yes | down |
| MsG0280006445.01 | -4.016195411 | 0.000556 | yes | down |
| MsG0280006446.01 | -2.917316462 | 6.45E-05 | yes | down |
| MsG0280006455.01 | -3.881477088 | 1.56E-05 | yes | down |
| MsG0280006457.01 | -3.867699457 | 6.61E-05 | yes | down |
| MsG0280006583.01 | -2.385153799 | 0.000181 | yes | down |
| MsG0280006695.01 | 1.603777824 | 3.21E-06 | yes | up |
| MsG0280006704.01 | -1.914921745 | 0.000813 | yes | down |
| MsG0280006737.01 | -1.724151002 | 0.0002 | yes | down |
| MsG0280006743.01 | 2.018862151 | 0.000247 | yes | up |
| MsG0280006768.01 | 2.03471235 | 4.63E-05 | yes | up |
| MsG0280006794.01 | -3.23248919 | 0.001207 | yes | down |
| MsG0280006855.01 | -3.133080404 | 0.000306 | yes | down |
| MsG0280006882.01 | -6.608519723 | 0.001171 | yes | down |
| MsG0280007050.01 | -1.916681987 | 4.78E-06 | yes | down |
| MsG0280007054.01 | -5.445923887 | 0.001491 | yes | down |
| MsG0280007182.01 | 1.023782743 | 0.001749 | yes | up |
| MsG0280007201.01 | -4.694579235 | 3.89E-07 | yes | down |
| MsG0280007391.01 | -2.645186378 | 2.34E-06 | yes | down |
| MsG0280007423.01 | 1.493670813 | 0.001006 | yes | up |
| MsG0280007547.01 | -4.216365688 | 8.55E-06 | yes | down |
| MsG0280007584.01 | -3.004677046 | 0.001215 | yes | down |
| MsG0280007643.01 | 7.506667845 | 5.58E-05 | yes | up |
| MsG0280007722.01 | -1.353968089 | 8.15E-05 | yes | down |
| MsG0280007759.01 | -6.503506948 | 0.000176 | yes | down |
| MsG0280007763.01 | -1.624295439 | 0.000812 | yes | down |
| MsG0280007789.01 | 1.581696212 | 0.00077 | yes | up |
| MsG0280007869.01 | -3.606378244 | 0.000105 | yes | down |
| MsG0280007906.01 | -6.176662219 | 4.38E-11 | yes | down |
| MsG0280007907.01 | -6.797826848 | 0.000162 | yes | down |
| MsG0280007908.01 | -6.042679406 | 1.16E-13 | yes | down |
| MsG0280007909.01 | -5.749283815 | 1.97E-05 | yes | down |
| MsG0280007910.01 | -5.660352451 | 2.51E-06 | yes | down |
| MsG0280007911.01 | -5.014384194 | 3.02E-08 | yes | down |
| MsG0280007934.01 | -4.386874794 | 0.000807 | yes | down |
| MsG0280007997.01 | -4.91451858 | 0.000395 | yes | down |
| MsG0280008038.01 | -3.521405439 | 4.22E-06 | yes | down |
| MsG0280008323.01 | -7.069534337 | 0.000186 | yes | down |
| MsG0280008349.01 | 3.428464315 | 1.21E-09 | yes | up |
| MsG0280008350.01 | 4.124096923 | 5.66E-10 | yes | up |
| MsG0280008352.01 | 2.825179899 | 7.80E-07 | yes | up |
| MsG0280008357.01 | 2.396985976 | 0.000514 | yes | up |
| MsG0280008405.01 | -4.426270369 | 1.34E-18 | yes | down |
| MsG0280008407.01 | -1.976847893 | 0.001077 | yes | down |
| MsG0280008492.01 | -1.710398548 | 0.000906 | yes | down |
| MsG0280008551.01 | -2.329501197 | 0.000323 | yes | down |
| MsG0280008574.01 | 1.099510012 | 0.001638 | yes | up |
| MsG0280008576.01 | 1.93626167 | 0.00156 | yes | up |
| MsG0280008697.01 | -2.036241199 | 1.65E-06 | yes | down |
| MsG0280008860.01 | 2.038805537 | 0.00011 | yes | up |
| MsG0280009223.01 | -4.160531679 | 0.000218 | yes | down |
| MsG0280009291.01 | -2.496114055 | 4.33E-06 | yes | down |
| MsG0280009545.01 | -2.845788025 | 0.001096 | yes | down |
| MsG0280009553.01 | -1.810024835 | 0.000287 | yes | down |
| MsG0280009574.01 | -1.429538363 | 8.90E-05 | yes | down |
| MsG0280009636.01 | -5.28097725 | 0.000117 | yes | down |
| MsG0280009674.01 | -1.574018036 | 0.000148 | yes | down |
| MsG0280009735.01 | 4.064711831 | 0.00023 | yes | up |
| MsG0280009745.01 | -3.173922681 | 0.000981 | yes | down |
| MsG0280009855.01 | -2.369136985 | 2.97E-05 | yes | down |
| MsG0280009899.01 | -1.717664318 | 0.000121 | yes | down |
| MsG0280009941.01 | 5.902763526 | 0.000469 | yes | up |
| MsG0280009942.01 | -5.469000633 | 0.000155 | yes | down |
| MsG0280010047.01 | -2.687048311 | 0.001661 | yes | down |
| MsG0280010102.01 | -2.990396992 | 0.000264 | yes | down |
| MsG0280010426.01 | -2.28288655 | 4.22E-07 | yes | down |
| MsG0280010428.01 | -3.746132984 | 2.32E-05 | yes | down |
| MsG0280010569.01 | -6.208654362 | 0.00107 | yes | down |
| MsG0280010592.01 | -1.336517024 | 0.001243 | yes | down |
| MsG0280010672.01 | -5.410185588 | 0.00014 | yes | down |
| MsG0280010692.01 | -1.062988047 | 0.0012 | yes | down |
| MsG0280010730.01 | -1.916349784 | 0.000661 | yes | down |
| MsG0280010863.01 | 4.531956803 | 6.87E-05 | yes | up |
| MsG0280010892.01 | 6.132789042 | 0.001383 | yes | up |
| MsG0280010945.01 | 1.083372307 | 0.001797 | yes | up |
| MsG0280011116.01 | 1.208827973 | 0.001149 | yes | up |
| MsG0280011136.01 | -3.755305204 | 6.82E-06 | yes | down |
| MsG0280011140.01 | 1.543471982 | 0.000347 | yes | up |
| MsG0280011165.01 | -5.462325437 | 6.42E-05 | yes | down |
| MsG0280011167.01 | -1.090728879 | 0.001777 | yes | down |
| MsG0280011203.01 | -4.505079989 | 0.0002 | yes | down |
| MsG0280011238.01 | -3.596087181 | 0.001601 | yes | down |
| MsG0280011336.01 | -1.12575197 | 2.28E-05 | yes | down |
| MsG0280011465.01 | -1.790287658 | 4.92E-05 | yes | down |
| MsG0380011703.01 | -3.350944187 | 0.001712 | yes | down |
| MsG0380011798.01 | -5.219585536 | 1.47E-10 | yes | down |
| MsG0380011868.01 | -2.518114184 | 0.001478 | yes | down |
| MsG0380011901.01 | 1.843834624 | 0.001676 | yes | up |
| MsG0380012044.01 | -2.622712452 | 0.000936 | yes | down |
| MsG0380012057.01 | 2.686103895 | 7.61E-06 | yes | up |
| MsG0380012069.01 | -3.757653045 | 0.000592 | yes | down |
| MsG0380012099.01 | -3.978055692 | 0.000277 | yes | down |
| MsG0380012244.01 | -7.96178169 | 2.43E-11 | yes | down |
| MsG0380012249.01 | 1.876925467 | 0.000952 | yes | up |
| MsG0380012382.01 | -4.394925945 | 6.42E-05 | yes | down |
| MsG0380012410.01 | -5.236187428 | 0.000381 | yes | down |
| MsG0380012606.01 | -7.916233647 | 1.91E-05 | yes | down |
| MsG0380012814.01 | -5.87566822 | 0.001655 | yes | down |
| MsG0380012889.01 | 3.268619423 | 6.70E-05 | yes | up |
| MsG0380012921.01 | 5.913580561 | 0.000114 | yes | up |
| MsG0380013072.01 | 6.938924295 | 0.000135 | yes | up |
| MsG0380013073.01 | 6.816270059 | 3.23E-05 | yes | up |
| MsG0380013210.01 | 5.740644043 | 3.06E-07 | yes | up |
| MsG0380013212.01 | 9.191396898 | 2.00E-09 | yes | up |
| MsG0380013213.01 | 5.807863148 | 0.00018 | yes | up |
| MsG0380013214.01 | 8.889414675 | 1.02E-08 | yes | up |
| MsG0380013223.01 | 6.859170857 | 0.000647 | yes | up |
| MsG0380013239.01 | 6.403920486 | 1.28E-10 | yes | up |
| MsG0380013278.01 | -2.535857087 | 0.000525 | yes | down |
| MsG0380013300.01 | -1.7690485 | 0.001139 | yes | down |
| MsG0380013436.01 | -2.648589246 | 0.000344 | yes | down |
| MsG0380013832.01 | 2.733006192 | 0.001453 | yes | up |
| MsG0380014090.01 | -7.497573047 | 0.000628 | yes | down |
| MsG0380014117.01 | -2.737656536 | 9.40E-06 | yes | down |
| MsG0380014122.01 | -7.155583764 | 6.38E-05 | yes | down |
| MsG0380014264.01 | 3.84123122 | 0.000247 | yes | up |
| MsG0380014610.01 | -1.892338673 | 0.000127 | yes | down |
| MsG0380014615.01 | -2.536443113 | 0.000993 | yes | down |
| MsG0380014627.01 | 3.454248448 | 1.25E-06 | yes | up |
| MsG0380014733.01 | -6.857126603 | 4.70E-05 | yes | down |
| MsG0380014754.01 | -1.559507018 | 0.001301 | yes | down |
| MsG0380014771.01 | -4.494017659 | 0.000119 | yes | down |
| MsG0380014883.01 | 1.160791807 | 0.000772 | yes | up |
| MsG0380014903.01 | -2.386504525 | 0.000964 | yes | down |
| MsG0380014975.01 | -4.042381121 | 0.000996 | yes | down |
| MsG0380015008.01 | -3.768777674 | 1.96E-07 | yes | down |
| MsG0380015009.01 | -3.809467126 | 1.14E-06 | yes | down |
| MsG0380015347.01 | 3.09310943 | 3.21E-07 | yes | up |
| MsG0380015353.01 | 1.650694547 | 0.0004 | yes | up |
| MsG0380015369.01 | -2.033225849 | 8.32E-05 | yes | down |
| MsG0380015438.01 | 1.998587322 | 0.001335 | yes | up |
| MsG0380015456.01 | -3.462058149 | 0.000153 | yes | down |
| MsG0380015500.01 | 2.223313614 | 8.10E-05 | yes | up |
| MsG0380015543.01 | -5.605928791 | 3.63E-07 | yes | down |
| MsG0380015606.01 | -3.412623183 | 0.000452 | yes | down |
| MsG0380015633.01 | 3.097687535 | 0.001238 | yes | up |
| MsG0380015637.01 | -3.403273266 | 4.04E-05 | yes | down |
| MsG0380015694.01 | 3.687587572 | 9.47E-05 | yes | up |
| MsG0380015741.01 | -2.120479897 | 0.000421 | yes | down |
| MsG0380015761.01 | 1.577543226 | 0.001007 | yes | up |
| MsG0380015800.01 | -1.469927299 | 5.34E-05 | yes | down |
| MsG0380015805.01 | -2.303960573 | 1.13E-05 | yes | down |
| MsG0380015880.01 | -1.92330347 | 5.08E-06 | yes | down |
| MsG0380015944.01 | -3.252419275 | 0.001391 | yes | down |
| MsG0380016022.01 | -1.991519382 | 0.000109 | yes | down |
| MsG0380016036.01 | -1.227593638 | 0.000948 | yes | down |
| MsG0380016060.01 | -5.983952652 | 3.54E-05 | yes | down |
| MsG0380016077.01 | 6.187907647 | 0.000782 | yes | up |
| MsG0380016084.01 | 2.509127736 | 0.001475 | yes | up |
| MsG0380016086.01 | -3.906978786 | 9.84E-05 | yes | down |
| MsG0380016130.01 | 1.846259163 | 0.000189 | yes | up |
| MsG0380016169.01 | -3.158551614 | 3.81E-05 | yes | down |
| MsG0380016297.01 | -6.165841013 | 0.00093 | yes | down |
| MsG0380016458.01 | -3.485547201 | 4.50E-07 | yes | down |
| MsG0380016521.01 | -2.924623616 | 0.000533 | yes | down |
| MsG0380016572.01 | -1.630035348 | 0.000555 | yes | down |
| MsG0380016598.01 | -2.741940295 | 0.001209 | yes | down |
| MsG0380016624.01 | -6.755668962 | 2.09E-07 | yes | down |
| MsG0380016717.01 | -1.43699615 | 0.001702 | yes | down |
| MsG0380016801.01 | -2.107828941 | 0.000261 | yes | down |
| MsG0380016905.01 | -1.252276818 | 0.00134 | yes | down |
| MsG0380017151.01 | -4.249689474 | 0.001551 | yes | down |
| MsG0380017164.01 | -2.994380963 | 0.000927 | yes | down |
| MsG0380017174.01 | -1.675190081 | 0.000426 | yes | down |
| MsG0380017237.01 | -4.032750039 | 2.24E-07 | yes | down |
| MsG0380017239.01 | -4.733582473 | 4.48E-06 | yes | down |
| MsG0380017338.01 | -4.371146837 | 3.75E-07 | yes | down |
| MsG0380017368.01 | -6.009059396 | 2.06E-16 | yes | down |
| MsG0380017384.01 | 1.183391177 | 0.000136 | yes | up |
| MsG0380017404.01 | -2.354952783 | 0.00035 | yes | down |
| MsG0380017746.01 | -2.677637329 | 0.001648 | yes | down |
| MsG0380017747.01 | -1.585884847 | 0.000146 | yes | down |
| MsG0380017842.01 | -4.331079503 | 6.51E-10 | yes | down |
| MsG0380017883.01 | 1.904393183 | 4.44E-05 | yes | up |
| MsG0380017913.01 | -2.449013493 | 5.89E-06 | yes | down |
| MsG0380017993.01 | -5.535928614 | 0.000304 | yes | down |
| MsG0380018006.01 | -5.190943154 | 0.001121 | yes | down |
| MsG0480018132.01 | -3.041474813 | 4.65E-10 | yes | down |
| MsG0480018217.01 | -2.541961567 | 7.26E-07 | yes | down |
| MsG0480018236.01 | 1.483181543 | 8.64E-06 | yes | up |
| MsG0480018350.01 | -3.426381903 | 3.52E-08 | yes | down |
| MsG0480018352.01 | -2.642748132 | 0.00018 | yes | down |
| MsG0480018398.01 | 3.233694021 | 3.28E-05 | yes | up |
| MsG0480018434.01 | -5.803196377 | 0.000673 | yes | down |
| MsG0480018627.01 | 1.685366129 | 0.000536 | yes | up |
| MsG0480018648.01 | -2.163386386 | 0.000394 | yes | down |
| MsG0480018697.01 | -5.303419293 | 6.18E-07 | yes | down |
| MsG0480018769.01 | 2.790295249 | 2.57E-05 | yes | up |
| MsG0480018780.01 | -1.498795214 | 0.001166 | yes | down |
| MsG0480018847.01 | -4.59317155 | 4.48E-05 | yes | down |
| MsG0480018904.01 | -1.538993761 | 0.001567 | yes | down |
| MsG0480019007.01 | 1.724834531 | 3.52E-05 | yes | up |
| MsG0480019019.01 | -1.865228118 | 0.000578 | yes | down |
| MsG0480019038.01 | -2.830288749 | 9.09E-07 | yes | down |
| MsG0480019214.01 | -6.279603787 | 0.001686 | yes | down |
| MsG0480019217.01 | -5.463870049 | 6.58E-06 | yes | down |
| MsG0480019295.01 | -2.099363332 | 0.000112 | yes | down |
| MsG0480019379.01 | 7.63415729 | 7.25E-08 | yes | up |
| MsG0480019510.01 | -5.915960665 | 0.000502 | yes | down |
| MsG0480019595.01 | 6.494127333 | 0.001286 | yes | up |
| MsG0480019669.01 | -1.171635821 | 0.000118 | yes | down |
| MsG0480019705.01 | -8.397703105 | 1.07E-08 | yes | down |
| MsG0480019879.01 | -1.620155973 | 0.001513 | yes | down |
| MsG0480019880.01 | -2.192991885 | 3.70E-05 | yes | down |
| MsG0480019881.01 | -3.160464979 | 2.73E-09 | yes | down |
| MsG0480020242.01 | -3.189659421 | 6.78E-05 | yes | down |
| MsG0480020251.01 | 1.358005954 | 0.001085 | yes | up |
| MsG0480020259.01 | 2.748667712 | 0.000324 | yes | up |
| MsG0480020263.01 | -2.173277947 | 0.000453 | yes | down |
| MsG0480020315.01 | -7.467321846 | 0.001613 | yes | down |
| MsG0480020319.01 | 4.485170207 | 0.000581 | yes | up |
| MsG0480020428.01 | -3.128080931 | 2.15E-07 | yes | down |
| MsG0480020445.01 | -2.433861478 | 0.000246 | yes | down |
| MsG0480020487.01 | -2.689665186 | 3.44E-07 | yes | down |
| MsG0480020488.01 | -3.497471511 | 0.000625 | yes | down |
| MsG0480020497.01 | -3.427672058 | 0.000285 | yes | down |
| MsG0480020534.01 | -1.210067468 | 0.000619 | yes | down |
| MsG0480020595.01 | -1.924850804 | 0.000121 | yes | down |
| MsG0480020814.01 | -3.537956799 | 0.001105 | yes | down |
| MsG0480020883.01 | 1.081270659 | 0.000726 | yes | up |
| MsG0480020915.01 | -2.997471841 | 1.79E-08 | yes | down |
| MsG0480020924.01 | -2.343368856 | 0.000252 | yes | down |
| MsG0480020934.01 | 6.092505369 | 2.50E-17 | yes | up |
| MsG0480020965.01 | -3.975557326 | 7.90E-08 | yes | down |
| MsG0480021198.01 | -3.067009681 | 0.000523 | yes | down |
| MsG0480021199.01 | -3.631849056 | 9.58E-05 | yes | down |
| MsG0480021200.01 | -3.872599619 | 7.02E-07 | yes | down |
| MsG0480021224.01 | -6.323066706 | 0.001768 | yes | down |
| MsG0480021379.01 | 3.089903937 | 0.001172 | yes | up |
| MsG0480021422.01 | 3.894833626 | 0.000222 | yes | up |
| MsG0480021471.01 | 6.0879664 | 3.59E-06 | yes | up |
| MsG0480021765.01 | -1.971610865 | 0.00012 | yes | down |
| MsG0480021789.01 | -5.64708048 | 2.19E-05 | yes | down |
| MsG0480021968.01 | 1.828315682 | 0.000156 | yes | up |
| MsG0480022036.01 | -2.527198247 | 0.001504 | yes | down |
| MsG0480022125.01 | -4.175993487 | 4.05E-09 | yes | down |
| MsG0480022156.01 | -4.005238169 | 0.000164 | yes | down |
| MsG0480022236.01 | 1.362405179 | 0.00174 | yes | up |
| MsG0480022241.01 | 1.306004537 | 0.000483 | yes | up |
| MsG0480022258.01 | 2.292166257 | 6.72E-07 | yes | up |
| MsG0480022311.01 | -3.903126294 | 6.42E-05 | yes | down |
| MsG0480022319.01 | -2.143071756 | 0.00024 | yes | down |
| MsG0480022375.01 | -1.795383524 | 0.000287 | yes | down |
| MsG0480022381.01 | 11.32887714 | 1.16E-07 | yes | up |
| MsG0480022481.01 | 1.295198529 | 0.000983 | yes | up |
| MsG0480022611.01 | -2.168605197 | 3.82E-05 | yes | down |
| MsG0480022655.01 | -4.997847006 | 1.21E-06 | yes | down |
| MsG0480022664.01 | 1.684474645 | 0.001673 | yes | up |
| MsG0480022702.01 | -2.073081287 | 4.65E-05 | yes | down |
| MsG0480022999.01 | -1.275784711 | 0.001462 | yes | down |
| MsG0480023001.01 | -2.120235031 | 9.05E-05 | yes | down |
| MsG0480023004.01 | -2.929171272 | 0.000841 | yes | down |
| MsG0480023109.01 | -2.383344201 | 0.001125 | yes | down |
| MsG0480023348.01 | -1.563440269 | 0.001548 | yes | down |
| MsG0480023371.01 | -1.335115899 | 0.001418 | yes | down |
| MsG0480023459.01 | -6.774061581 | 0.000844 | yes | down |
| MsG0480023541.01 | -1.05314672 | 0.001301 | yes | down |
| MsG0480023544.01 | 3.287781199 | 3.32E-06 | yes | up |
| MsG0480023620.01 | -2.454945402 | 0.001407 | yes | down |
| MsG0480023648.01 | -1.588932581 | 0.000138 | yes | down |
| MsG0480023658.01 | -1.659735205 | 4.87E-06 | yes | down |
| MsG0480023682.01 | -4.827660638 | 0.000128 | yes | down |
| MsG0480023690.01 | 1.099803157 | 0.001321 | yes | up |
| MsG0480023877.01 | 1.362244401 | 9.28E-05 | yes | up |
| MsG0480023878.01 | 1.378772075 | 0.001388 | yes | up |
| MsG0480023913.01 | -6.074002298 | 0.00085 | yes | down |
| MsG0480023920.01 | -2.601786048 | 9.67E-05 | yes | down |
| MsG0480023927.01 | -4.123794264 | 0.001822 | yes | down |
| MsG0480024012.01 | -1.697009932 | 0.000234 | yes | down |
| MsG0480024019.01 | -3.614858262 | 6.02E-05 | yes | down |
| MsG0580024083.01 | -3.818704731 | 0.000462 | yes | down |
| MsG0580024126.01 | -1.968869696 | 0.000569 | yes | down |
| MsG0580024154.01 | -2.915399595 | 4.02E-05 | yes | down |
| MsG0580024176.01 | -4.22412621 | 7.63E-08 | yes | down |
| MsG0580024178.01 | -3.678162206 | 2.24E-06 | yes | down |
| MsG0580024217.01 | -5.925996351 | 0.00067 | yes | down |
| MsG0580024220.01 | -6.172467557 | 3.94E-09 | yes | down |
| MsG0580024260.01 | -5.132218434 | 3.04E-06 | yes | down |
| MsG0580024321.01 | -5.095830551 | 0.001403 | yes | down |
| MsG0580024347.01 | 1.925425233 | 7.00E-06 | yes | up |
| MsG0580024408.01 | -4.990188584 | 3.88E-06 | yes | down |
| MsG0580024415.01 | -3.369636586 | 3.12E-10 | yes | down |
| MsG0580024435.01 | 2.113990043 | 0.000162 | yes | up |
| MsG0580024538.01 | 1.753206078 | 0.001311 | yes | up |
| MsG0580024546.01 | 1.232009898 | 0.000826 | yes | up |
| MsG0580024606.01 | 2.274235092 | 0.000277 | yes | up |
| MsG0580024756.01 | -2.282363538 | 0.001562 | yes | down |
| MsG0580024796.01 | -5.132009515 | 2.62E-05 | yes | down |
| MsG0580024805.01 | 1.308192083 | 6.62E-05 | yes | up |
| MsG0580024887.01 | -2.783203774 | 0.000212 | yes | down |
| MsG0580024957.01 | 2.942565081 | 1.02E-06 | yes | up |
| MsG0580024986.01 | -3.949739586 | 5.01E-06 | yes | down |
| MsG0580024990.01 | -4.790973395 | 1.66E-06 | yes | down |
| MsG0580025011.01 | 1.65835537 | 0.000162 | yes | up |
| MsG0580025041.01 | -7.194696961 | 0.001467 | yes | down |
| MsG0580025113.01 | -5.296448779 | 0.00015 | yes | down |
| MsG0580025157.01 | -4.507140606 | 1.86E-06 | yes | down |
| MsG0580025206.01 | 1.985072104 | 8.20E-05 | yes | up |
| MsG0580025224.01 | -5.406858239 | 4.73E-06 | yes | down |
| MsG0580025225.01 | -3.487705571 | 5.82E-09 | yes | down |
| MsG0580025275.01 | -2.451696251 | 0.000291 | yes | down |
| MsG0580025276.01 | -2.425677679 | 0.000776 | yes | down |
| MsG0580025280.01 | 1.803738578 | 5.30E-05 | yes | up |
| MsG0580025288.01 | -3.895405088 | 2.29E-05 | yes | down |
| MsG0580025313.01 | -2.48137387 | 0.001106 | yes | down |
| MsG0580025399.01 | -3.61073602 | 7.88E-05 | yes | down |
| MsG0580025444.01 | -3.973253293 | 0.001233 | yes | down |
| MsG0580025449.01 | -7.158580786 | 0.001314 | yes | down |
| MsG0580025523.01 | -3.662104811 | 1.36E-08 | yes | down |
| MsG0580025532.01 | -7.018010274 | 7.06E-06 | yes | down |
| MsG0580025576.01 | -6.278988259 | 0.001207 | yes | down |
| MsG0580025604.01 | -3.790208097 | 0.000334 | yes | down |
| MsG0580025858.01 | -5.588214077 | 4.99E-14 | yes | down |
| MsG0580025860.01 | -5.237407296 | 0.000392 | yes | down |
| MsG0580025893.01 | -2.379224089 | 0.001013 | yes | down |
| MsG0580025902.01 | -1.789955747 | 0.000224 | yes | down |
| MsG0580025906.01 | 1.408094173 | 0.001667 | yes | up |
| MsG0580025965.01 | -4.404960276 | 0.000584 | yes | down |
| MsG0580025987.01 | -3.292884225 | 0.000367 | yes | down |
| MsG0580026019.01 | -4.629506471 | 2.85E-08 | yes | down |
| MsG0580026027.01 | -4.627921204 | 0.000275 | yes | down |
| MsG0580026224.01 | 2.918407094 | 4.40E-05 | yes | up |
| MsG0580026269.01 | 1.168495651 | 0.000526 | yes | up |
| MsG0580026496.01 | 1.194441397 | 0.000371 | yes | up |
| MsG0580026524.01 | -6.412956747 | 0.000986 | yes | down |
| MsG0580026778.01 | -5.934669696 | 0.000111 | yes | down |
| MsG0580027033.01 | -4.883688044 | 2.26E-07 | yes | down |
| MsG0580027162.01 | -8.77619604 | 7.63E-12 | yes | down |
| MsG0580027191.01 | -5.662516632 | 1.81E-06 | yes | down |
| MsG0580027436.01 | -2.489102485 | 1.23E-05 | yes | down |
| MsG0580027607.01 | -2.172396003 | 0.001134 | yes | down |
| MsG0580027615.01 | 2.666069166 | 0.000141 | yes | up |
| MsG0580027617.01 | -4.529245989 | 3.23E-08 | yes | down |
| MsG0580027638.01 | -3.245897632 | 4.98E-08 | yes | down |
| MsG0580027733.01 | -2.273909623 | 0.000365 | yes | down |
| MsG0580027880.01 | -2.034929136 | 1.11E-05 | yes | down |
| MsG0580027929.01 | -1.825667446 | 0.001505 | yes | down |
| MsG0580027951.01 | 1.985487747 | 0.001152 | yes | up |
| MsG0580028076.01 | -7.769133225 | 2.86E-07 | yes | down |
| MsG0580028518.01 | 2.226188386 | 0.001792 | yes | up |
| MsG0580028529.01 | 6.524798525 | 0.000334 | yes | up |
| MsG0580028552.01 | 2.598429301 | 0.001784 | yes | up |
| MsG0580028553.01 | 2.878444758 | 5.20E-05 | yes | up |
| MsG0580028567.01 | -7.017034249 | 0.000115 | yes | down |
| MsG0580028603.01 | -1.679076439 | 0.000309 | yes | down |
| MsG0580028668.01 | -3.653159276 | 1.71E-05 | yes | down |
| MsG0580028708.01 | -4.589230148 | 6.47E-07 | yes | down |
| MsG0580028759.01 | -1.544428937 | 0.00138 | yes | down |
| MsG0580028840.01 | -2.814093957 | 0.000467 | yes | down |
| MsG0580028924.01 | -3.212242011 | 0.000195 | yes | down |
| MsG0580028988.01 | -2.394748916 | 0.000189 | yes | down |
| MsG0580029012.01 | -3.540912767 | 1.14E-05 | yes | down |
| MsG0580029101.01 | -3.908331155 | 4.13E-07 | yes | down |
| MsG0580029136.01 | 1.711759822 | 0.001555 | yes | up |
| MsG0580029251.01 | -2.033178602 | 2.94E-05 | yes | down |
| MsG0580029291.01 | -2.209390457 | 0.001086 | yes | down |
| MsG0580029296.01 | -4.358964051 | 2.65E-09 | yes | down |
| MsG0580029311.01 | 2.327110873 | 0.000865 | yes | up |
| MsG0580029333.01 | 2.37570805 | 0.000331 | yes | up |
| MsG0580029414.01 | 2.54614363 | 7.68E-06 | yes | up |
| MsG0580029667.01 | -2.887397738 | 0.000257 | yes | down |
| MsG0580029670.01 | -1.415743057 | 0.000958 | yes | down |
| MsG0580029730.01 | -7.961767745 | 6.97E-06 | yes | down |
| MsG0580029751.01 | 1.64847547 | 0.000548 | yes | up |
| MsG0580029805.01 | -2.74410482 | 7.51E-06 | yes | down |
| MsG0580030019.01 | -2.616852607 | 1.97E-08 | yes | down |
| MsG0580030055.01 | 2.046959787 | 0.000431 | yes | up |
| MsG0580030102.01 | -2.130423139 | 0.000712 | yes | down |
| MsG0580030181.01 | -2.707682976 | 0.001372 | yes | down |
| MsG0680030288.01 | -1.532357478 | 0.001382 | yes | down |
| MsG0680030296.01 | -2.122290347 | 0.001237 | yes | down |
| MsG0680030303.01 | -3.450813489 | 2.09E-07 | yes | down |
| MsG0680030358.01 | -3.910085081 | 0.000929 | yes | down |
| MsG0680030379.01 | -7.207346399 | 2.92E-06 | yes | down |
| MsG0680030380.01 | -5.463331918 | 9.12E-07 | yes | down |
| MsG0680030382.01 | -6.933464116 | 7.31E-07 | yes | down |
| MsG0680030391.01 | -6.674416397 | 4.63E-05 | yes | down |
| MsG0680030545.01 | 1.503092694 | 0.000158 | yes | up |
| MsG0680030554.01 | 3.353858567 | 0.001048 | yes | up |
| MsG0680030574.01 | -3.859332603 | 1.25E-10 | yes | down |
| MsG0680030613.01 | -2.660566068 | 0.000308 | yes | down |
| MsG0680030628.01 | -6.993384644 | 7.12E-05 | yes | down |
| MsG0680030651.01 | 2.117207235 | 3.69E-06 | yes | up |
| MsG0680030677.01 | -3.212816544 | 0.00069 | yes | down |
| MsG0680030746.01 | -1.607513039 | 0.001565 | yes | down |
| MsG0680030842.01 | 1.586686907 | 0.001053 | yes | up |
| MsG0680030857.01 | 2.050107379 | 0.001078 | yes | up |
| MsG0680030955.01 | -7.091241735 | 6.70E-05 | yes | down |
| MsG0680030982.01 | -3.580733522 | 0.001566 | yes | down |
| MsG0680030996.01 | -3.395865547 | 4.25E-05 | yes | down |
| MsG0680031139.01 | -3.853160597 | 3.77E-05 | yes | down |
| MsG0680031261.01 | -2.09052402 | 9.77E-08 | yes | down |
| MsG0680031429.01 | -3.877333223 | 0.000372 | yes | down |
| MsG0680031444.01 | 5.697500912 | 7.32E-06 | yes | up |
| MsG0680031678.01 | -4.321013956 | 0.001701 | yes | down |
| MsG0680031835.01 | -3.394861334 | 3.92E-11 | yes | down |
| MsG0680031843.01 | -3.107709687 | 1.41E-05 | yes | down |
| MsG0680031962.01 | 2.92528577 | 0.001088 | yes | up |
| MsG0680031993.01 | 2.141764785 | 0.001367 | yes | up |
| MsG0680032019.01 | -1.952428987 | 3.88E-06 | yes | down |
| MsG0680032026.01 | -3.274244984 | 4.25E-05 | yes | down |
| MsG0680032027.01 | -3.039913925 | 0.000125 | yes | down |
| MsG0680032054.01 | -3.193537968 | 2.08E-06 | yes | down |
| MsG0680032221.01 | 2.951709087 | 9.23E-05 | yes | up |
| MsG0680032264.01 | 4.921983137 | 0.000983 | yes | up |
| MsG0680032274.01 | 3.173032471 | 0.001255 | yes | up |
| MsG0680032285.01 | 3.933097569 | 0.000534 | yes | up |
| MsG0680032290.01 | 3.855451196 | 0.001152 | yes | up |
| MsG0680032301.01 | 3.736293528 | 0.000766 | yes | up |
| MsG0680032302.01 | 2.627603388 | 0.000147 | yes | up |
| MsG0680032310.01 | 4.016839915 | 0.000193 | yes | up |
| MsG0680032431.01 | 3.231039365 | 3.68E-07 | yes | up |
| MsG0680032461.01 | -4.066110265 | 2.46E-08 | yes | down |
| MsG0680032533.01 | 6.647489542 | 4.36E-09 | yes | up |
| MsG0680032954.01 | 2.941454984 | 2.63E-06 | yes | up |
| MsG0680033136.01 | -4.461664151 | 0.000506 | yes | down |
| MsG0680033339.01 | 1.083316625 | 0.001649 | yes | up |
| MsG0680033393.01 | 3.648302122 | 1.81E-08 | yes | up |
| MsG0680034192.01 | -5.15677429 | 0.00041 | yes | down |
| MsG0680034221.01 | -3.843974284 | 6.35E-05 | yes | down |
| MsG0680034255.01 | 4.360789272 | 1.39E-07 | yes | up |
| MsG0680034313.01 | 3.407998843 | 0.000806 | yes | up |
| MsG0680034317.01 | 4.200437306 | 3.75E-09 | yes | up |
| MsG0680034489.01 | 2.081571618 | 0.000996 | yes | up |
| MsG0680034708.01 | -2.417855311 | 0.000777 | yes | down |
| MsG0680034996.01 | 6.891830943 | 0.000915 | yes | up |
| MsG0680035094.01 | 5.400669954 | 0.000477 | yes | up |
| MsG0680035153.01 | 5.739828077 | 9.62E-11 | yes | up |
| MsG0680035158.01 | 3.827187673 | 0.00056 | yes | up |
| MsG0680035364.01 | -2.992329379 | 9.60E-06 | yes | down |
| MsG0680035568.01 | -1.625444183 | 0.000645 | yes | down |
| MsG0680035779.01 | -3.588688203 | 7.20E-05 | yes | down |
| MsG0680035780.01 | -2.003435887 | 0.001309 | yes | down |
| MsG0680035781.01 | -1.370920425 | 0.000182 | yes | down |
| MsG0680035855.01 | 1.181896939 | 0.001022 | yes | up |
| MsG0780035923.01 | -2.049006124 | 6.20E-05 | yes | down |
| MsG0780036078.01 | -2.560444558 | 0.000625 | yes | down |
| MsG0780036333.01 | 7.283324079 | 0.001154 | yes | up |
| MsG0780036334.01 | 7.667204487 | 7.02E-05 | yes | up |
| MsG0780036411.01 | -3.90315645 | 2.55E-13 | yes | down |
| MsG0780036552.01 | 1.641698471 | 0.000146 | yes | up |
| MsG0780036562.01 | -4.099795555 | 0.001043 | yes | down |
| MsG0780036661.01 | -2.418298806 | 2.59E-06 | yes | down |
| MsG0780036818.01 | -2.981441824 | 5.84E-06 | yes | down |
| MsG0780036949.01 | -1.192661952 | 0.001427 | yes | down |
| MsG0780036976.01 | -7.305030169 | 2.44E-05 | yes | down |
| MsG0780036981.01 | -5.057229393 | 4.27E-06 | yes | down |
| MsG0780036982.01 | -5.372130552 | 2.77E-10 | yes | down |
| MsG0780036983.01 | -7.018315077 | 5.69E-10 | yes | down |
| MsG0780036984.01 | -7.337211594 | 1.34E-17 | yes | down |
| MsG0780037004.01 | -2.799855455 | 3.44E-06 | yes | down |
| MsG0780037103.01 | -5.097357192 | 0.000311 | yes | down |
| MsG0780037105.01 | 1.948727751 | 0.000358 | yes | up |
| MsG0780037235.01 | -1.978906405 | 5.28E-05 | yes | down |
| MsG0780037381.01 | -2.85215605 | 0.00011 | yes | down |
| MsG0780037395.01 | -2.139531364 | 0.000409 | yes | down |
| MsG0780037414.01 | -2.70030221 | 0.000618 | yes | down |
| MsG0780037701.01 | 1.267978634 | 0.000803 | yes | up |
| MsG0780037943.01 | -5.325413806 | 0.000836 | yes | down |
| MsG0780038116.01 | -3.183195548 | 9.81E-05 | yes | down |
| MsG0780038123.01 | -2.771653782 | 0.000136 | yes | down |
| MsG0780038132.01 | -3.563643645 | 4.86E-06 | yes | down |
| MsG0780038135.01 | -3.162538571 | 2.24E-06 | yes | down |
| MsG0780038329.01 | -7.223743844 | 0.001507 | yes | down |
| MsG0780038393.01 | -1.687724569 | 0.000499 | yes | down |
| MsG0780038399.01 | -1.383943074 | 0.001073 | yes | down |
| MsG0780038430.01 | -1.809770232 | 0.000201 | yes | down |
| MsG0780038459.01 | 4.699971769 | 2.77E-05 | yes | up |
| MsG0780038465.01 | 2.681603055 | 0.001343 | yes | up |
| MsG0780038471.01 | 2.261964693 | 0.000246 | yes | up |
| MsG0780038515.01 | -2.460953397 | 1.01E-05 | yes | down |
| MsG0780038662.01 | -3.369615908 | 5.90E-06 | yes | down |
| MsG0780038860.01 | -2.905883272 | 0.000449 | yes | down |
| MsG0780038914.01 | 3.023346052 | 0.000841 | yes | up |
| MsG0780038919.01 | 3.220452029 | 5.52E-07 | yes | up |
| MsG0780038923.01 | -3.132442146 | 0.000136 | yes | down |
| MsG0780038925.01 | -3.291142699 | 5.68E-05 | yes | down |
| MsG0780038956.01 | -1.763937607 | 5.74E-06 | yes | down |
| MsG0780039104.01 | -4.173966776 | 1.13E-05 | yes | down |
| MsG0780039131.01 | -3.99052669 | 0.000615 | yes | down |
| MsG0780039134.01 | -5.015331389 | 0.001176 | yes | down |
| MsG0780039188.01 | -1.308820033 | 0.001195 | yes | down |
| MsG0780039288.01 | -4.91658036 | 7.04E-08 | yes | down |
| MsG0780039303.01 | -2.275747065 | 2.85E-05 | yes | down |
| MsG0780039356.01 | -2.30016823 | 0.0006 | yes | down |
| MsG0780039503.01 | -3.407244984 | 0.00055 | yes | down |
| MsG0780039544.01 | -3.207218959 | 2.48E-06 | yes | down |
| MsG0780039576.01 | -3.541799517 | 4.19E-05 | yes | down |
| MsG0780039580.01 | -2.829963072 | 4.88E-05 | yes | down |
| MsG0780039591.01 | 5.295280983 | 0.000988 | yes | up |
| MsG0780039706.01 | 1.20247116 | 0.001538 | yes | up |
| MsG0780039877.01 | -1.422761346 | 0.000904 | yes | down |
| MsG0780039968.01 | -3.125918816 | 0.000843 | yes | down |
| MsG0780039973.01 | 2.453399064 | 0.000434 | yes | up |
| MsG0780039989.01 | -2.218391589 | 8.66E-05 | yes | down |
| MsG0780040005.01 | -2.568753936 | 0.001825 | yes | down |
| MsG0780040096.01 | -2.039327988 | 0.00012 | yes | down |
| MsG0780040114.01 | -3.015180435 | 0.000125 | yes | down |
| MsG0780040116.01 | -3.297330221 | 4.64E-07 | yes | down |
| MsG0780040185.01 | -4.384639907 | 3.25E-06 | yes | down |
| MsG0780040276.01 | -1.800564886 | 0.000922 | yes | down |
| MsG0780040294.01 | -3.839050264 | 2.65E-05 | yes | down |
| MsG0780040335.01 | -1.668472267 | 8.49E-06 | yes | down |
| MsG0780040374.01 | 1.012718078 | 0.001515 | yes | up |
| MsG0780040428.01 | 1.296333159 | 0.000571 | yes | up |
| MsG0780040439.01 | 2.238407798 | 6.86E-07 | yes | up |
| MsG0780040440.01 | 1.853802428 | 0.000143 | yes | up |
| MsG0780040460.01 | -2.536790903 | 0.001747 | yes | down |
| MsG0780040480.01 | -3.320600789 | 0.000187 | yes | down |
| MsG0780040519.01 | -2.330279452 | 2.05E-08 | yes | down |
| MsG0780040676.01 | -2.940795024 | 0.000689 | yes | down |
| MsG0780040711.01 | -5.209201618 | 1.22E-05 | yes | down |
| MsG0780040806.01 | -4.453970968 | 0.000722 | yes | down |
| MsG0780040942.01 | -3.046257226 | 3.08E-10 | yes | down |
| MsG0780040943.01 | -3.056080975 | 1.26E-07 | yes | down |
| MsG0780040945.01 | -2.201958798 | 0.00033 | yes | down |
| MsG0780040952.01 | -2.671386718 | 8.26E-08 | yes | down |
| MsG0780040954.01 | -8.723318041 | 1.78E-08 | yes | down |
| MsG0780040959.01 | -11.71545032 | 4.20E-19 | yes | down |
| MsG0780040961.01 | -9.532126049 | 6.50E-11 | yes | down |
| MsG0780041023.01 | -3.16879277 | 0.000516 | yes | down |
| MsG0780041042.01 | -2.470124821 | 2.59E-06 | yes | down |
| MsG0780041062.01 | -2.192686276 | 0.00011 | yes | down |
| MsG0780041069.01 | 5.393904989 | 1.80E-05 | yes | up |
| MsG0780041072.01 | -3.15327595 | 6.16E-05 | yes | down |
| MsG0780041102.01 | -3.356321217 | 0.001234 | yes | down |
| MsG0780041201.01 | 1.625567129 | 0.00059 | yes | up |
| MsG0780041250.01 | -1.541085218 | 0.00044 | yes | down |
| MsG0780041316.01 | -1.832984984 | 0.001363 | yes | down |
| MsG0780041327.01 | -2.890352803 | 0.000665 | yes | down |
| MsG0780041393.01 | -1.433903426 | 0.000711 | yes | down |
| MsG0780041556.01 | -3.242516992 | 9.40E-05 | yes | down |
| MsG0880041861.01 | -3.799560588 | 1.75E-06 | yes | down |
| MsG0880041871.01 | -1.327093129 | 2.60E-05 | yes | down |
| MsG0880042443.01 | 1.540574158 | 0.001584 | yes | up |
| MsG0880042531.01 | -1.564986471 | 0.000229 | yes | down |
| MsG0880042572.01 | -6.511353434 | 0.001 | yes | down |
| MsG0880042597.01 | -5.678356091 | 0.000902 | yes | down |
| MsG0880042633.01 | 1.455151547 | 0.001036 | yes | up |
| MsG0880042634.01 | -8.774515055 | 5.05E-08 | yes | down |
| MsG0880042644.01 | -2.560608094 | 0.00014 | yes | down |
| MsG0880042714.01 | -1.379608481 | 0.00018 | yes | down |
| MsG0880042745.01 | -1.53103021 | 0.001572 | yes | down |
| MsG0880042776.01 | 1.133640497 | 0.001374 | yes | up |
| MsG0880042836.01 | -2.967106216 | 3.90E-05 | yes | down |
| MsG0880042866.01 | -4.744335267 | 1.96E-07 | yes | down |
| MsG0880042918.01 | 4.706018725 | 5.54E-06 | yes | up |
| MsG0880042931.01 | 3.023833292 | 0.001224 | yes | up |
| MsG0880042933.01 | 2.459781393 | 4.50E-05 | yes | up |
| MsG0880042948.01 | -2.154730545 | 6.73E-05 | yes | down |
| MsG0880042953.01 | 1.811288408 | 5.24E-06 | yes | up |
| MsG0880043105.01 | 1.574819321 | 0.001214 | yes | up |
| MsG0880043258.01 | -4.102190148 | 7.01E-06 | yes | down |
| MsG0880043285.01 | -2.958697475 | 0.000322 | yes | down |
| MsG0880043286.01 | -2.151090216 | 0.000459 | yes | down |
| MsG0880043511.01 | -5.672333897 | 2.92E-15 | yes | down |
| MsG0880043536.01 | -2.405199341 | 0.000229 | yes | down |
| MsG0880043537.01 | -2.096910076 | 0.001045 | yes | down |
| MsG0880043808.01 | 1.410666664 | 0.001327 | yes | up |
| MsG0880043815.01 | 7.091440081 | 0.000348 | yes | up |
| MsG0880043854.01 | 2.33177835 | 0.000868 | yes | up |
| MsG0880043937.01 | 2.895984479 | 3.91E-05 | yes | up |
| MsG0880043959.01 | 1.184236104 | 0.001537 | yes | up |
| MsG0880043983.01 | 2.330996203 | 0.000887 | yes | up |
| MsG0880044007.01 | -3.334582021 | 3.77E-06 | yes | down |
| MsG0880044037.01 | -3.541940228 | 0.000228 | yes | down |
| MsG0880044055.01 | -2.193495555 | 0.000847 | yes | down |
| MsG0880044211.01 | 2.175352556 | 0.000325 | yes | up |
| MsG0880044228.01 | -1.474075576 | 0.000342 | yes | down |
| MsG0880044259.01 | -2.134454896 | 6.16E-06 | yes | down |
| MsG0880044284.01 | -2.957640229 | 2.31E-05 | yes | down |
| MsG0880044306.01 | -2.407308178 | 0.00163 | yes | down |
| MsG0880044312.01 | 2.362960029 | 0.000214 | yes | up |
| MsG0880044346.01 | 1.682941894 | 3.06E-09 | yes | up |
| MsG0880044349.01 | 3.684465304 | 0.000662 | yes | up |
| MsG0880044415.01 | 2.800162714 | 0.000151 | yes | up |
| MsG0880044416.01 | 1.966315786 | 0.001795 | yes | up |
| MsG0880044524.01 | 3.663951842 | 0.001035 | yes | up |
| MsG0880044656.01 | -5.837073007 | 1.58E-06 | yes | down |
| MsG0880044711.01 | -1.037909177 | 0.001126 | yes | down |
| MsG0880044813.01 | -2.333406825 | 0.001432 | yes | down |
| MsG0880044837.01 | -6.18257395 | 2.51E-20 | yes | down |
| MsG0880045275.01 | -2.020716128 | 1.27E-05 | yes | down |
| MsG0880045363.01 | 2.034062004 | 0.001274 | yes | up |
| MsG0880045441.01 | -2.014655479 | 4.82E-05 | yes | down |
| MsG0880045474.01 | -5.22471612 | 2.63E-05 | yes | down |
| MsG0880045537.01 | -2.483003499 | 0.000394 | yes | down |
| MsG0880045543.01 | 1.379469958 | 6.32E-05 | yes | up |
| MsG0880045645.01 | -2.778875288 | 7.60E-07 | yes | down |
| MsG0880045796.01 | -5.042050254 | 3.15E-11 | yes | down |
| MsG0880045907.01 | -1.567719827 | 0.000585 | yes | down |
| MsG0880045909.01 | -2.063066348 | 0.000486 | yes | down |
| MsG0880045915.01 | -1.394896619 | 0.001536 | yes | down |
| MsG0880045997.01 | -1.297964978 | 0.000246 | yes | down |
| MsG0880046012.01 | -1.635927637 | 0.000751 | yes | down |
| MsG0880046106.01 | 2.271963213 | 0.000268 | yes | up |
| MsG0880046140.01 | 4.05785502 | 3.56E-08 | yes | up |
| MsG0880046231.01 | -4.288112362 | 2.02E-06 | yes | down |
| MsG0880046293.01 | 1.33429216 | 0.000879 | yes | up |
| MsG0880046325.01 | -2.300913221 | 8.19E-05 | yes | down |
| MsG0880046428.01 | 1.686944263 | 0.000729 | yes | up |
| MsG0880046471.01 | 1.073940584 | 0.000126 | yes | up |
| MsG0880046528.01 | -1.920791934 | 9.63E-08 | yes | down |
| MsG0880046618.01 | -2.220574848 | 0.001531 | yes | down |
| MsG0880046740.01 | -4.93357055 | 5.93E-06 | yes | down |
| MsG0880046888.01 | -3.043985575 | 7.06E-07 | yes | down |
| MsG0880046908.01 | -4.057316526 | 4.52E-05 | yes | down |
| MsG0880046940.01 | -5.078110033 | 0.001099 | yes | down |
| MsG0880046950.01 | 3.247422456 | 4.47E-06 | yes | up |
| MsG0880046996.01 | -2.509734975 | 0.000548 | yes | down |
| MsG0880047084.01 | -4.707606755 | 2.26E-06 | yes | down |
| MsG0880047085.01 | -4.268082545 | 2.95E-05 | yes | down |
| MsG0880047191.01 | 2.012332739 | 2.48E-06 | yes | up |
| MsG0880047211.01 | -6.926604469 | 2.08E-05 | yes | down |
| MsG0880047228.01 | -2.651257113 | 0.000961 | yes | down |
| MsG0880047256.01 | -3.636015444 | 0.000956 | yes | down |
| MsG0880047296.01 | 1.587639955 | 4.39E-05 | yes | up |
| MsG0880047391.01 | 2.429229025 | 0.000475 | yes | up |
| MsG0880047484.01 | -3.02866439 | 2.63E-07 | yes | down |
| MsG0880047551.01 | 1.223245417 | 0.001523 | yes | up |
| MsG0880047563.01 | -1.991467338 | 0.000405 | yes | down |
| MsG0880047572.01 | -4.544438783 | 6.25E-05 | yes | down |
| MsG0880047581.01 | 2.877963645 | 1.69E-05 | yes | up |
| MsG0880047654.01 | -2.327287601 | 0.000805 | yes | down |
| MsG0880047747.01 | -1.872748988 | 2.05E-05 | yes | down |
| MsG0880047761.01 | 4.653229854 | 3.75E-06 | yes | up |
| MsG0880047777.01 | -1.286747899 | 0.000881 | yes | down |
