# Supplementary material for: Analysis of physiological characteristics and gene co-expression networks in Medicago sativa roots under low-temperature stress
Source: Front Plant Sci. 2025 Aug 25;16:1597949. doi: 10.3389/fpls.2025.1597949 (PMC12415042; doi:10.3389/fpls.2025.1597949)
Supplement: Supplementary file 3 [file Table2.docx]

**GO functional annotations and the number of differentially expressed genes statistics**

| **GO ID** | **GO Term** | **Term Type** | **Longmu801_**  **vs_ Sardi**  **number** | **Longmu801_vs_Sardi percent** |
| --- | --- | --- | --- | --- |
| GO:0065007 | biological regulation | biological_process | 28 | 28/ 150 |
| GO:0008152 | metabolic process | biological_process | 65 | 65/ 150 |
| GO:0022414 | reproductive process | biological_process | 2 | 2/ 150 |
| GO:0000003 | reproduction | biological_process | 1 | 1/ 150 |
| GO:0009987 | cellular process | biological_process | 75 | 75/ 150 |
| GO:0032502 | developmental process | biological_process | 3 | 3/ 150 |
| GO:0044419 | biological process involved in interspecies interaction between organisms | biological_process | 2 | 2/ 150 |
| GO:0040007 | growth | biological_process | 1 | 1/ 150 |
| GO:0051179 | localization | biological_process | 8 | 8/ 150 |
| GO:0050896 | response to stimulus | biological_process | 31 | 31/ 150 |
| GO:0032991 | protein-containing complex | cellular_component | 4 | 4/ 150 |
| GO:0110165 | cellular anatomical entity | cellular_component | 85 | 85/ 150 |
| GO:0045182 | translation regulator activity | molecular_function | 2 | 2/ 150 |
| GO:0140110 | transcription regulator activity | molecular_function | 11 | 11/ 150 |
| GO:0005198 | structural molecule activity | molecular_function | 4 | 4/ 150 |
| GO:0140657 | ATP-dependent activity | molecular_function | 3 | 3/ 150 |
| GO:0044183 | protein folding chaperone | molecular_function | 1 | 1/ 150 |
| GO:0016209 | antioxidant activity | molecular_function | 5 | 5/ 150 |
| GO:0005215 | transporter activity | molecular_function | 6 | 6/ 150 |
| GO:0098772 | molecular function regulator activity | molecular_function | 6 | 6/ 150 |
| GO:0005488 | binding | molecular_function | 75 | 75/ 150 |
| GO:0045735 | nutrient reservoir activity | molecular_function | 1 | 1/ 150 |
| GO:0060089 | molecular transducer activity | molecular_function | 3 | 3/ 150 |
| GO:0003824 | catalytic activity | molecular_function | 85 | 85/ 150 |
