# Supplementary material for: Analysis of physiological characteristics and gene co-expression networks in Medicago sativa roots under low-temperature stress
Source: Front Plant Sci. 2025 Aug 25;16:1597949. doi: 10.3389/fpls.2025.1597949 (PMC12415042; doi:10.3389/fpls.2025.1597949)
Supplement: Supplementary file 4 [file Table3.docx]

**GO enrichment analysis of differential expressed genes in the root of Longmu801_vs_ Sardi alfalfa**

| **GO** **ID** | **Term Type** | **Description** | **Number** | **Rich factor** | **P-value** | **Gene ID** |
| --- | --- | --- | --- | --- | --- | --- |
| GO:0009699 | BP | phenylpropanoid biosynthetic process | 7 | 0.046358 | 2.80E-07 | MsG0780040942.01;MsG0180005357.01;MsG0580025604.01;MsG0880045441.01;MsG0780040943.01;MsG0780040945.01;MsG0180003237.01 |
| GO:0009698 | BP | phenylpropanoid metabolic process | 7 | 0.039773 | 7.88E-07 | MsG0780040942.01;MsG0180005357.01;MsG0580025604.01;MsG0880045441.01;MsG0780040943.01;MsG0780040945.01;MsG0180003237.01 |
| GO:0044550 | BP | secondary metabolite biosynthetic process | 7 | 0.039106 | 8.83E-07 | MsG0780040942.01;MsG0180005357.01;MsG0580025604.01;MsG0880045441.01;MsG0780040943.01;MsG0780040945.01;MsG0180003237.01 |
| GO:0005199 | MF | structural constituent of cell wall | 4 | 0.16 | 7.51E-07 | MsG0680032019.01;MsG0180005143.01;MsG0180005130.01;MsG0680032026.01 |
| GO:0006558 | BP | L-phenylalanine metabolic process | 4 | 0.129032 | 1.84E-06 | MsG0780040942.01;MsG0480020595.01;MsG0780040943.01;MsG0780040945.01 |
| GO:1902221 | BP | erythrose 4-phosphate/phosphoenolpyruvate family amino acid metabolic process | 4 | 0.129032 | 1.84E-06 | MsG0780040942.01;MsG0480020595.01;MsG0780040943.01;MsG0780040945.01 |
| GO:0120255 | BP | olefinic compound biosynthetic process | 4 | 0.111111 | 3.41E-06 | MsG0780040942.01;MsG0180005357.01;MsG0780040943.01;MsG0780040945.01 |
| GO:0120254 | BP | olefinic compound metabolic process | 4 | 0.105263 | 4.26E-06 | MsG0780040942.01;MsG0180005357.01;MsG0780040943.01;MsG0780040945.01 |
| GO:0009755 | BP | hormone-mediated signaling pathway | 12 | 0.014545 | 4.59E-06 | MsG0880047085.01;MsG0780039188.01;MsG0580025288.01;MsG0580025225.01;MsG0180001649.01;MsG0280007908.01;MsG0880047084.01;MsG0380011868.01;MsG0280006794.01;MsG0180001655.01;MsG0780036818.01;MsG0180004830.01 |
| GO:0009800 | BP | cinnamic acid biosynthetic process | 3 | 0.214286 | 7.96E-06 | MsG0780040942.01;MsG0780040943.01;MsG0780040945.01 |
| GO:0009803 | BP | cinnamic acid metabolic process | 3 | 0.214286 | 7.96E-06 | MsG0780040942.01;MsG0780040943.01;MsG0780040945.01 |
| GO:0042537 | BP | benzene-containing compound metabolic process | 4 | 0.086957 | 9.25E-06 | MsG0780040942.01;MsG0780040943.01;MsG0080048387.01;MsG0780040945.01 |
| GO:0009664 | BP | plant-type cell wall organization | 4 | 0.083333 | 1.10E-05 | MsG0680032019.01;MsG0180005143.01;MsG0180005130.01;MsG0680032026.01 |
| GO:0019748 | BP | secondary metabolic process | 7 | 0.024648 | 1.82E-05 | MsG0780040942.01;MsG0180005357.01;MsG0580025604.01;MsG0880045441.01;MsG0780040943.01;MsG0780040945.01;MsG0180003237.01 |
| GO:0043178 | MF | alcohol binding | 4 | 0.072727 | 1.90E-05 | MsG0280007908.01;MsG0880047084.01;MsG0580026224.01;MsG0880047085.01 |
| GO:0045548 | MF | phenylalanine ammonia-lyase activity | 3 | 0.15 | 2.46E-05 | MsG0780040942.01;MsG0780040943.01;MsG0780040945.01 |
| GO:0006559 | BP | L-phenylalanine catabolic process | 3 | 0.125 | 4.34E-05 | MsG0780040942.01;MsG0780040943.01;MsG0780040945.01 |
| GO:1902222 | BP | erythrose 4-phosphate/phosphoenolpyruvate family amino acid catabolic process | 3 | 0.125 | 4.34E-05 | MsG0780040942.01;MsG0780040943.01;MsG0780040945.01 |
| GO:0008194 | MF | UDP-glycosyltransferase activity | 9 | 0.014493 | 7.63E-05 | MsG0580025858.01;MsG0280009291.01;MsG0680032054.01;MsG0780041023.01;MsG0780038116.01;MsG0780038135.01;MsG0780038123.01;MsG0780039877.01;MsG0780039356.01 |
| GO:0016758 | MF | hexosyltransferase activity | 10 | 0.012642 | 9.41E-05 | MsG0580025858.01;MsG0880047484.01;MsG0280009291.01;MsG0680032054.01;MsG0780041023.01;MsG0780038116.01;MsG0780038135.01;MsG0780038123.01;MsG0780039877.01;MsG0780039356.01 |
| GO:0007165 | BP | signal transduction | 18 | 0.007647 | 0.0001 | MsG0280009553.01;MsG0880047085.01;MsG0780039188.01;MsG0580025288.01;MsG0580025225.01;MsG0180001649.01;MsG0280007908.01;MsG0880047084.01;MsG0380011868.01;MsG0580026496.01;MsG0380015543.01;MsG0580029805.01;MsG0280006794.01;MsG0880042597.01;MsG0180001655.01;MsG0780036818.01;MsG0180004830.01;MsG0580026019.01 |
| GO:0005576 | CC | extracellular region | 11 | 0.011329 | 0.0001 | MsG0880042836.01;MsG0580027615.01;MsG0880046106.01;MsG0280010428.01;MsG0280008407.01;MsG0280009899.01;MsG0180003237.01;MsG0680030382.01;MsG0580027607.01;MsG0580029291.01;MsG0480019881.01 |
| GO:0016841 | MF | ammonia-lyase activity | 3 | 0.090909 | 0.0001 | MsG0780040942.01;MsG0780040943.01;MsG0780040945.01 |
| GO:0080043 | MF | quercetin 3-O-glucosyltransferase activity | 4 | 0.044944 | 0.0001 | MsG0280009291.01;MsG0580025858.01;MsG0780039877.01;MsG0780039356.01 |
| GO:0080044 | MF | quercetin 7-O-glucosyltransferase activity | 4 | 0.044944 | 0.0001 | MsG0280009291.01;MsG0580025858.01;MsG0780039877.01;MsG0780039356.01 |
| GO:0080163 | BP | regulation of protein serine/threonine phosphatase activity | 3 | 0.085714 | 0.0001 | MsG0280007908.01;MsG0880047084.01;MsG0880047085.01 |
| GO:0009072 | BP | aromatic amino acid family metabolic process | 4 | 0.042553 | 0.0001 | MsG0780040942.01;MsG0480020595.01;MsG0780040943.01;MsG0780040945.01 |
| GO:0010427 | MF | abscisic acid binding | 3 | 0.076923 | 0.0001 | MsG0280007908.01;MsG0880047084.01;MsG0880047085.01 |
| GO:0009074 | BP | aromatic amino acid family catabolic process | 3 | 0.075 | 0.0002 | MsG0780040942.01;MsG0780040943.01;MsG0780040945.01 |
| GO:0043666 | BP | regulation of phosphoprotein phosphatase activity | 3 | 0.073171 | 0.0002 | MsG0280007908.01;MsG0880047084.01;MsG0880047085.01 |
| GO:0006749 | BP | glutathione metabolic process | 4 | 0.038095 | 0.0002 | MsG0180004941.01;MsG0480023109.01;MsG0380015009.01;MsG0380015008.01 |
| GO:0042562 | MF | hormone binding | 3 | 0.071429 | 0.0002 | MsG0280007908.01;MsG0880047084.01;MsG0880047085.01 |
| GO:0019840 | MF | isoprenoid binding | 3 | 0.071429 | 0.0002 | MsG0280007908.01;MsG0880047084.01;MsG0880047085.01 |
| GO:0010921 | BP | regulation of phosphatase activity | 3 | 0.065217 | 0.0003 | MsG0280007908.01;MsG0880047084.01;MsG0880047085.01 |
| GO:0004364 | MF | glutathione transferase activity | 4 | 0.035088 | 0.0003 | MsG0180004941.01;MsG0480023109.01;MsG0380015009.01;MsG0380015008.01 |
| GO:0035304 | BP | regulation of protein dephosphorylation | 3 | 0.061224 | 0.0003 | MsG0280007908.01;MsG0880047084.01;MsG0880047085.01 |
| GO:0004864 | MF | protein phosphatase inhibitor activity | 3 | 0.061224 | 0.0003 | MsG0280007908.01;MsG0880047084.01;MsG0880047085.01 |
| GO:0019212 | MF | phosphatase inhibitor activity | 3 | 0.058824 | 0.0004 | MsG0280007908.01;MsG0880047084.01;MsG0880047085.01 |
| GO:0035303 | BP | regulation of dephosphorylation | 3 | 0.05 | 0.00068 | MsG0280007908.01;MsG0880047084.01;MsG0880047085.01 |
| GO:0016757 | MF | glycosyltransferase activity | 11 | 0.009106 | 0.0006 | MsG0580025858.01;MsG0880047484.01;MsG0580025902.01;MsG0280009291.01;MsG0680032054.01;MsG0780041023.01;MsG0780038116.01;MsG0780038135.01;MsG0780038123.01;MsG0780039877.01;MsG0780039356.01 |
| GO:0071555 | BP | cell wall organization | 6 | 0.016043 | 0.0007 | MsG0880042836.01;MsG0680032026.01;MsG0680032019.01;MsG0780041316.01;MsG0180005143.01;MsG0180005130.01 |
| GO:0009738 | BP | abscisic acid-activated signaling pathway | 3 | 0.047619 | 0.0007 | MsG0280007908.01;MsG0880047084.01;MsG0880047085.01 |
| GO:0009873 | BP | ethylene-activated signaling pathway | 3 | 0.046875 | 0.0008 | MsG0280006794.01;MsG0780036818.01;MsG0180004830.01 |
| GO:0071669 | BP | plant-type cell wall organization or biogenesis | 4 | 0.024845 | 0.0011 | MsG0680032019.01;MsG0180005143.01;MsG0180005130.01;MsG0680032026.01 |
| GO:0035251 | MF | UDP-glucosyltransferase activity | 5 | 0.018116 | 0.0011 | MsG0780038116.01;MsG0580025858.01;MsG0280009291.01;MsG0780039877.01;MsG0780039356.01 |
| GO:0033293 | MF | monocarboxylic acid binding | 3 | 0.041096 | 0.0012 | MsG0280007908.01;MsG0880047084.01;MsG0880047085.01 |
| GO:0045229 | BP | external encapsulating structure organization | 6 | 0.014458 | 0.0012 | MsG0880042836.01;MsG0680032026.01;MsG0680032019.01;MsG0780041316.01;MsG0180005143.01;MsG0180005130.01 |
| GO:0016209 | MF | antioxidant activity | 5 | 0.017182 | 0.0015 | MsG0180004941.01;MsG0480023541.01;MsG0180001149.01;MsG0580029291.01;MsG0280010428.01 |
| GO:0016840 | MF | carbon-nitrogen lyase activity | 3 | 0.037037 | 0.0016 | MsG0780040942.01;MsG0780040943.01;MsG0780040945.01 |
| GO:0006575 | BP | cellular modified amino acid metabolic process | 4 | 0.021622 | 0.0019 | MsG0180004941.01;MsG0480023109.01;MsG0380015009.01;MsG0380015008.01 |
| GO:0019888 | MF | protein phosphatase regulator activity | 3 | 0.033708 | 0.0021 | MsG0280007908.01;MsG0880047084.01;MsG0880047085.01 |
| GO:0071554 | BP | cell wall organization or biogenesis | 6 | 0.012903 | 0.0022 | MsG0880042836.01;MsG0680032026.01;MsG0680032019.01;MsG0780041316.01;MsG0180005143.01;MsG0180005130.01 |
| GO:0019208 | MF | phosphatase regulator activity | 3 | 0.032967 | 0.0022 | MsG0280007908.01;MsG0880047084.01;MsG0880047085.01 |
| GO:0051336 | BP | regulation of hydrolase activity | 3 | 0.032609 | 0.0023 | MsG0280007908.01;MsG0880047084.01;MsG0880047085.01 |
| GO:0046527 | MF | glucosyltransferase activity | 5 | 0.015244 | 0.0025 | MsG0780038116.01;MsG0580025858.01;MsG0280009291.01;MsG0780039877.01;MsG0780039356.01 |
| GO:0016052 | BP | carbohydrate catabolic process | 6 | 0.012474 | 0.00261 | MsG0380012889.01;MsG0780041062.01;MsG0280011136.01;MsG0880042836.01;MsG0780041316.01;MsG0580027615.01 |
| GO:0000272 | BP | polysaccharide catabolic process | 5 | 0.015106 | 0.0026 | MsG0780041316.01;MsG0380012889.01;MsG0280011136.01;MsG0580027615.01;MsG0880042836.01 |
| GO:0016491 | MF | oxidoreductase activity | 19 | 0.005719 | 0.0034 | MsG0080048598.01;MsG0280010428.01;MsG0580024178.01;MsG0380011901.01;MsG0180002286.01;MsG0380014615.01;MsG0180004941.01;MsG0180004203.01;MsG0480023541.01;MsG0580025604.01;MsG0380014883.01;MsG0880042633.01;MsG0380015606.01;MsG0880045441.01;MsG0680032221.01;MsG0180001149.01;MsG0880045275.01;MsG0580029291.01;MsG0480020445.01 |
| GO:0043177 | MF | organic acid binding | 3 | 0.028302 | 0.0034 | MsG0280007908.01;MsG0880047084.01;MsG0880047085.01 |
| GO:0032787 | BP | monocarboxylic acid metabolic process | 6 | 0.011472 | 0.00392 | MsG0780040942.01;MsG0780041062.01;MsG0780040943.01;MsG0080048387.01;MsG0780040945.01;MsG0880042633.01 |
| GO:0050896 | BP | response to stimulus | 31 | 0.004678 | 0.0041 | MsG0280011136.01;MsG0880043286.01;MsG0880047777.01;MsG0480023109.01;MsG0680033393.01;MsG0880043285.01;MsG0880044346.01;MsG0380015008.01;MsG0180003462.01;MsG0880043983.01;MsG0180004941.01;MsG0080048598.01;MsG0280010428.01;MsG0280008407.01;MsG0780039989.01;MsG0380015009.01;MsG0580026496.01;MsG0880042776.01;MsG0880042597.01;MsG0480023878.01;MsG0280007908.01;MsG0880042948.01;MsG0580024083.01;MsG0880047085.01;MsG0580029311.01;MsG0380015543.01;MsG0880047084.01;MsG0580026224.01;MsG0380015761.01;MsG0880043959.01;MsG0580029291.01 |
| GO:0016684 | MF | oxidoreductase activity, acting on peroxide as acceptor | 4 | 0.017316 | 0.0043 | MsG0280010428.01;MsG0480023541.01;MsG0180001149.01;MsG0580029291.01 |
| GO:0004601 | MF | peroxidase activity | 4 | 0.017316 | 0.0043 | MsG0280010428.01;MsG0480023541.01;MsG0180001149.01;MsG0580029291.01 |
| GO:0031406 | MF | carboxylic acid binding | 3 | 0.025641 | 0.0045 | MsG0280007908.01;MsG0880047084.01;MsG0880047085.01 |
| GO:0004857 | MF | enzyme inhibitor activity | 6 | 0.011029 | 0.0047 | MsG0880042836.01;MsG0880047085.01;MsG0280009899.01;MsG0280007908.01;MsG0880047084.01;MsG0780041316.01 |
| GO:0004476 | MF | mannose-6-phosphate isomerase activity | 1 | 0.5 | 0.0056 | MsG0780036078.01 |
| GO:0080046 | MF | quercetin 4'-O-glucosyltransferase activity | 1 | 0.5 | 0.0056 | MsG0280009291.01 |
| GO:0045727 | BP | positive regulation of translation | 2 | 0.045455 | 0.0069 | MsG0580026269.01;MsG0280009674.01 |
| GO:0034250 | BP | positive regulation of cellular amide metabolic process | 2 | 0.045455 | 0.0069 | MsG0580026269.01;MsG0280009674.01 |
| GO:0072330 | BP | monocarboxylic acid biosynthetic process | 4 | 0.015094 | 0.00706 | MsG0880042633.01;MsG0780040942.01;MsG0780040943.01;MsG0780040945.01 |
| GO:0042221 | BP | response to chemical | 8 | 0.008147 | 0.00718 | MsG0380015008.01;MsG0180004941.01;MsG0480023109.01;MsG0380015009.01;MsG0680033393.01;MsG0580026496.01;MsG0880042948.01;MsG0380015761.01 |
| GO:0003700 | MF | DNA-binding transcription factor activity | 11 | 0.006724 | 0.0071 | MsG0380017239.01;MsG0780039288.01;MsG0280007391.01;MsG0280006743.01;MsG0880042953.01;MsG0580025532.01;MsG0580027951.01;MsG0280006794.01;MsG0780036818.01;MsG0180004830.01;MsG0780038399.01 |
| GO:0016671 | MF | oxidoreductase activity, acting on a sulfur group of donors, disulfide as acceptor | 2 | 0.041667 | 0.0082 | MsG0380014883.01;MsG0180002286.01 |
| GO:0050373 | MF | UDP-arabinose 4-epimerase activity | 1 | 0.333333 | 0.0084 | MsG0280007789.01 |
| GO:0055114 | BP | obsolete oxidation-reduction process | 11 | 0.006505 | 0.0090 | MsG0280010428.01;MsG0580024178.01;MsG0380011901.01;MsG0380014615.01;MsG0180004203.01;MsG0480023541.01;MsG0580025604.01;MsG0880042633.01;MsG0380015606.01;MsG0580029291.01;MsG0480020445.01 |
| GO:0009063 | BP | cellular amino acid catabolic process | 3 | 0.019481 | 0.0097 | MsG0780040942.01;MsG0780040943.01;MsG0780040945.01 |
| GO:0016998 | BP | cell wall macromolecule catabolic process | 2 | 0.037736 | 0.0100 | MsG0280011136.01;MsG0580027615.01 |
| GO:0050794 | BP | regulation of cellular process | 28 | 0.004554 | 0.0101 | MsG0280009553.01;MsG0280007391.01;MsG0580025288.01;MsG0280007908.01;MsG0580026269.01;MsG0280006794.01;MsG0780036818.01;MsG0880042953.01;MsG0580025532.01;MsG0180001655.01;MsG0180001649.01;MsG0380017239.01;MsG0780038399.01;MsG0780039188.01;MsG0580026496.01;MsG0880042776.01;MsG0580029805.01;MsG0880042597.01;MsG0180004830.01;MsG0780039288.01;MsG0880047085.01;MsG0280006743.01;MsG0580025225.01;MsG0880047084.01;MsG0380011868.01;MsG0280009674.01;MsG0380015543.01;MsG0580026019.01 |
| GO:1901606 | BP | alpha-amino acid catabolic process | 3 | 0.018987 | 0.0104 | MsG0780040942.01;MsG0780040943.01;MsG0780040945.01 |
| GO:0051246 | BP | regulation of protein metabolic process | 5 | 0.010799 | 0.0105 | MsG0280007908.01;MsG0880047084.01;MsG0580026269.01;MsG0280009674.01;MsG0880047085.01 |
| GO:0046209 | BP | nitric oxide metabolic process | 1 | 0.25 | 0.0113 | MsG0380015606.01 |
| GO:0006809 | BP | nitric oxide biosynthetic process | 1 | 0.25 | 0.0113 | MsG0380015606.01 |
| GO:0006452 | BP | translational frameshifting | 1 | 0.25 | 0.0113 | MsG0580026269.01 |
| GO:0009360 | CC | DNA polymerase III complex | 1 | 0.25 | 0.0113 | MsG0880047777.01 |
| GO:1990137 | MF | plant seed peroxidase activity | 1 | 0.25 | 0.0113 | MsG0180001149.01 |
| GO:0102070 | MF | 18-hydroxyoleate peroxygenase activity | 1 | 0.25 | 0.0113 | MsG0180001149.01 |
| GO:0050464 | MF | nitrate reductase (NADPH) activity | 1 | 0.25 | 0.0113 | MsG0380015606.01 |
| GO:0010628 | BP | positive regulation of gene expression | 2 | 0.034483 | 0.0119 | MsG0580026269.01;MsG0280009674.01 |
| GO:0016667 | MF | oxidoreductase activity, acting on a sulfur group of donors | 3 | 0.017964 | 0.0121 | MsG0180004941.01;MsG0380014883.01;MsG0180002286.01 |
| GO:0019220 | BP | regulation of phosphate metabolic process | 3 | 0.017857 | 0.0123 | MsG0280007908.01;MsG0880047084.01;MsG0880047085.01 |
| GO:0051174 | BP | regulation of phosphorus metabolic process | 3 | 0.017857 | 0.0123 | MsG0280007908.01;MsG0880047084.01;MsG0880047085.01 |
| GO:0097305 | BP | response to alcohol | 2 | 0.033333 | 0.0127 | MsG0580026496.01;MsG0680033393.01 |
| GO:0009737 | BP | response to abscisic acid | 2 | 0.033333 | 0.0127 | MsG0580026496.01;MsG0680033393.01 |
| GO:0005976 | BP | polysaccharide metabolic process | 6 | 0.008876 | 0.0130 | MsG0380012889.01;MsG0280011136.01;MsG0880042836.01;MsG0780041316.01;MsG0380016036.01;MsG0580027615.01 |
| GO:0000160 | BP | phosphorelay signal transduction system | 3 | 0.017442 | 0.0131 | MsG0280006794.01;MsG0780036818.01;MsG0180004830.01 |
| GO:0006952 | BP | defense response | 12 | 0.00616 | 0.0132 | MsG0880043285.01;MsG0280011136.01;MsG0880043286.01;MsG0880047085.01;MsG0880043983.01;MsG0580024083.01;MsG0280007908.01;MsG0880047084.01;MsG0380015543.01;MsG0880043959.01;MsG0180003462.01;MsG0880042597.01 |
| GO:0045901 | BP | positive regulation of translational elongation | 1 | 0.2 | 0.0141 | MsG0580026269.01 |
| GO:0009298 | BP | GDP-mannose biosynthetic process | 1 | 0.2 | 0.0141 | MsG0780036078.01 |
| GO:0004664 | MF | prephenate dehydratase activity | 1 | 0.2 | 0.0141 | MsG0480020595.01 |
| GO:0004465 | MF | lipoprotein lipase activity | 1 | 0.2 | 0.0141 | MsG0180003862.01 |
| GO:0047769 | MF | arogenate dehydratase activity | 1 | 0.2 | 0.0141 | MsG0480020595.01 |
| GO:0005737 | CC | cytoplasm | 19 | 0.004969 | 0.0163 | MsG0180005354.01;MsG0780040942.01;MsG0880047085.01;MsG0780040943.01;MsG0180004941.01;MsG0480023109.01;MsG0780041062.01;MsG0380015009.01;MsG0780040945.01;MsG0280007908.01;MsG0880047084.01;MsG0880042633.01;MsG0580026224.01;MsG0380015761.01;MsG0380015008.01;MsG0280009553.01;MsG0580027607.01;MsG0480020595.01;MsG0480020445.01 |
| GO:0035970 | BP | peptidyl-threonine dephosphorylation | 2 | 0.028986 | 0.0165 | MsG0080048230.01;MsG0580024347.01 |
| GO:0045905 | BP | positive regulation of translational termination | 1 | 0.166667 | 0.0169 | MsG0580026269.01 |
| GO:0098599 | MF | palmitoyl hydrolase activity | 1 | 0.166667 | 0.0169 | MsG0580024546.01 |
| GO:0043546 | MF | molybdopterin cofactor binding | 1 | 0.166667 | 0.0169 | MsG0380015606.01 |
| GO:0047837 | MF | D-xylose 1-dehydrogenase (NADP+) activity | 1 | 0.166667 | 0.0169 | MsG0680032221.01 |
| GO:0031399 | BP | regulation of protein modification process | 3 | 0.015385 | 0.01834 | MsG0280007908.01;MsG0880047084.01;MsG0880047085.01 |
| GO:1902223 | BP | erythrose 4-phosphate/phosphoenolpyruvate family amino acid biosynthetic process | 1 | 0.142857 | 0.0197 | MsG0480020595.01 |
| GO:0019673 | BP | GDP-mannose metabolic process | 1 | 0.142857 | 0.0197 | MsG0780036078.01 |
| GO:0009094 | BP | L-phenylalanine biosynthetic process | 1 | 0.142857 | 0.0197 | MsG0480020595.01 |
| GO:0043243 | BP | positive regulation of protein-containing complex disassembly | 1 | 0.125 | 0.0224 | MsG0580026269.01 |
| GO:0006449 | BP | regulation of translational termination | 1 | 0.125 | 0.0224 | MsG0580026269.01 |
| GO:0000492 | BP | box C/D snoRNP assembly | 1 | 0.125 | 0.0224 | MsG0280009574.01 |
| GO:0051177 | BP | meiotic sister chromatid cohesion | 1 | 0.125 | 0.0224 | MsG0380016905.01 |
| GO:0046395 | BP | carboxylic acid catabolic process | 3 | 0.013953 | 0.0236 | MsG0780040942.01;MsG0780040943.01;MsG0780040945.01 |
| GO:0046394 | BP | carboxylic acid biosynthetic process | 5 | 0.008389 | 0.0280 | MsG0880042633.01;MsG0780040942.01;MsG0780040943.01;MsG0480020595.01;MsG0780040945.01 |
| GO:0000491 | BP | small nucleolar ribonucleoprotein complex assembly | 1 | 0.1 | 0.0280 | MsG0280009574.01 |
| GO:0016054 | BP | organic acid catabolic process | 3 | 0.012987 | 0.0284 | MsG0780040942.01;MsG0780040943.01;MsG0780040945.01 |
| GO:0044283 | BP | small molecule biosynthetic process | 6 | 0.007282 | 0.0306 | MsG0780040942.01;MsG0480020595.01;MsG0780040943.01;MsG0780040945.01;MsG0880042633.01;MsG0180005357.01 |
| GO:0019953 | BP | sexual reproduction | 1 | 0.090909 | 0.0307 | MsG0880046106.01 |
| GO:0009714 | BP | chalcone metabolic process | 1 | 0.090909 | 0.0307 | MsG0180005357.01 |
| GO:0009715 | BP | chalcone biosynthetic process | 1 | 0.090909 | 0.0307 | MsG0180005357.01 |
| GO:1901605 | BP | alpha-amino acid metabolic process | 4 | 0.009479 | 0.0326 | MsG0780040942.01;MsG0780040943.01;MsG0480020595.01;MsG0780040945.01 |
| GO:0016053 | BP | organic acid biosynthetic process | 5 | 0.008026 | 0.0329 | MsG0880042633.01;MsG0780040942.01;MsG0780040943.01;MsG0480020595.01;MsG0780040945.01 |
| GO:0006790 | BP | sulfur compound metabolic process | 4 | 0.009368 | 0.0339 | MsG0180004941.01;MsG0480023109.01;MsG0380015009.01;MsG0380015008.01 |
| GO:0043436 | BP | oxoacid metabolic process | 8 | 0.006102 | 0.0339 | MsG0780040942.01;MsG0480020595.01;MsG0780041062.01;MsG0780040943.01;MsG0080048387.01;MsG0780040945.01;MsG0880042633.01;MsG0380015606.01 |
| GO:0033993 | BP | response to lipid | 2 | 0.019608 | 0.0342 | MsG0580026496.01;MsG0680033393.01 |
| GO:0050789 | BP | regulation of biological process | 28 | 0.004138 | 0.0361 | MsG0280009553.01;MsG0280007391.01;MsG0580025288.01;MsG0280007908.01;MsG0580026269.01;MsG0280006794.01;MsG0780036818.01;MsG0880042953.01;MsG0580025532.01;MsG0180001655.01;MsG0180001649.01;MsG0380017239.01;MsG0780038399.01;MsG0780039188.01;MsG0580026496.01;MsG0880042776.01;MsG0580029805.01;MsG0880042597.01;MsG0180004830.01;MsG0780039288.01;MsG0880047085.01;MsG0280006743.01;MsG0580025225.01;MsG0880047084.01;MsG0380011868.01;MsG0280009674.01;MsG0380015543.01;MsG0580026019.01 |
| GO:0009095 | BP | aromatic amino acid family biosynthetic process, prephenate pathway | 1 | 0.076923 | 0.0362 | MsG0480020595.01 |
| GO:0019438 | BP | aromatic compound biosynthetic process | 10 | 0.005624 | 0.0364 | MsG0180001447.01;MsG0780040942.01;MsG0180005357.01;MsG0580025604.01;MsG0880045441.01;MsG0780040943.01;MsG0780040945.01;MsG0780036078.01;MsG0180003237.01;MsG0480020595.01 |
| GO:2001057 | BP | reactive nitrogen species metabolic process | 1 | 0.071429 | 0.0390 | MsG0380015606.01 |
| GO:0045903 | BP | positive regulation of translational fidelity | 1 | 0.071429 | 0.0390 | MsG0280009674.01 |
| GO:0009959 | BP | negative gravitropism | 1 | 0.071429 | 0.0390 | MsG0880044346.01 |
| GO:0042126 | BP | nitrate metabolic process | 1 | 0.071429 | 0.0390 | MsG0380015606.01 |
| GO:0042128 | BP | nitrate assimilation | 1 | 0.071429 | 0.0390 | MsG0380015606.01 |
| GO:0006646 | BP | phosphatidylethanolamine biosynthetic process | 1 | 0.066667 | 0.0417 | MsG0180005354.01 |
| GO:0009696 | BP | salicylic acid metabolic process | 1 | 0.066667 | 0.0417 | MsG0080048387.01 |
| GO:0016477 | BP | cell migration | 1 | 0.066667 | 0.041746 | MsG0580027607.01 |
| GO:0000003 | BP | reproduction | 1 | 0.066667 | 0.0417 | MsG0880046106.01 |
| GO:0046337 | BP | phosphatidylethanolamine metabolic process | 1 | 0.066667 | 0.0417 | MsG0180005354.01 |
| GO:0048870 | BP | cell motility | 1 | 0.058824 | 0.0471 | MsG0580027607.01 |
| GO:0018958 | BP | phenol-containing compound metabolic process | 1 | 0.058824 | 0.0471 | MsG0080048387.01 |
| GO:0010088 | BP | phloem development | 1 | 0.055556 | 0.0498 | MsG0180004094.01 |
| GO:0031222 | BP | arabinan catabolic process | 1 | 0.055556 | 0.0498 | MsG0580027615.01 |
| GO:0031221 | BP | arabinan metabolic process | 1 | 0.055556 | 0.0498 | MsG0580027615.01 |
| GO:0005850 | CC | eukaryotic translation initiation factor 2 complex | 1 | 0.1 | 0.0280 | MsG0280009674.01 |
| GO:0005663 | CC | DNA replication factor C complex | 1 | 0.066667 | 0.0417 | MsG0880047777.01 |
| GO:0005794 | CC | Golgi apparatus | 4 | 0.008547 | 0.0449 | MsG0180002421.01;MsG0880046325.01;MsG0580026224.01;MsG0580025902.01 |
| GO:0048046 | CC | apoplast | 3 | 0.010453 | 0.0487 | MsG0280008407.01;MsG0680030382.01;MsG0180003237.01 |
| GO:0016672 | MF | oxidoreductase activity, acting on a sulfur group of donors, quinone or similar compound as acceptor | 1 | 0.142857 | 0.0197 | MsG0180004941.01 |
| GO:0045174 | MF | glutathione dehydrogenase (ascorbate) activity | 1 | 0.142857 | 0.0197 | MsG0180004941.01 |
| GO:0004174 | MF | electron-transferring-flavoprotein dehydrogenase activity | 1 | 0.142857 | 0.0197 | MsG0480020445.01 |
| GO:0008999 | MF | peptide-alanine-alpha-N-acetyltransferase activity | 1 | 0.125 | 0.0224 | MsG0580024154.01 |
| GO:0004103 | MF | choline kinase activity | 1 | 0.125 | 0.0224 | MsG0180005354.01 |
| GO:0004737 | MF | pyruvate decarboxylase activity | 1 | 0.125 | 0.0224 | MsG0080048597.01 |
| GO:0003978 | MF | UDP-glucose 4-epimerase activity | 1 | 0.111111 | 0.0252 | MsG0280007789.01 |
| GO:0016649 | MF | oxidoreductase activity, acting on the CH-NH group of donors, quinone or similar compound as acceptor | 1 | 0.111111 | 0.0252 | MsG0480020445.01 |
| GO:0140110 | MF | transcription regulator activity | 11 | 0.005644 | 0.0269 | MsG0380017239.01;MsG0780039288.01;MsG0280007391.01;MsG0280006743.01;MsG0880042953.01;MsG0580025532.01;MsG0580027951.01;MsG0280006794.01;MsG0780036818.01;MsG0180004830.01;MsG0780038399.01 |
| GO:0016765 | MF | transferase activity, transferring alkyl or aryl (other than methyl) groups | 4 | 0.009975 | 0.0278 | MsG0180004941.01;MsG0480023109.01;MsG0380015009.01;MsG0380015008.01 |
| GO:0004305 | MF | ethanolamine kinase activity | 1 | 0.1 | 0.0280 | MsG0180005354.01 |
| GO:0009703 | MF | nitrate reductase (NADH) activity | 1 | 0.090909 | 0.0307 | MsG0380015606.01 |
| GO:0008940 | MF | nitrate reductase activity | 1 | 0.090909 | 0.0307 | MsG0380015606.01 |
| GO:0000822 | MF | inositol hexakisphosphate binding | 1 | 0.090909 | 0.0307 | MsG0580026224.01 |
| GO:0050463 | MF | nitrate reductase [NAD(P)H] activity | 1 | 0.090909 | 0.0307 | MsG0380015606.01 |
| GO:0015114 | MF | phosphate ion transmembrane transporter activity | 1 | 0.090909 | 0.0307 | MsG0580026224.01 |
| GO:0000287 | MF | magnesium ion binding | 3 | 0.012448 | 0.0316 | MsG0080048597.01;MsG0780041062.01;MsG0880045275.01 |
| GO:0102406 | MF | omega-hydroxypalmitate O-sinapoyl transferase activity | 1 | 0.083333 | 0.0335 | MsG0380015500.01 |
| GO:1990136 | MF | linoleate 9S-lipoxygenase activity | 1 | 0.083333 | 0.0335 | MsG0880042633.01 |
| GO:0050660 | MF | flavin adenine dinucleotide binding | 3 | 0.012 | 0.0346 | MsG0380015606.01;MsG0380011901.01;MsG0480020445.01 |
| GO:0016629 | MF | 12-oxophytodienoate reductase activity | 1 | 0.076923 | 0.0362 | MsG0580024178.01 |
| GO:0030151 | MF | molybdenum ion binding | 1 | 0.076923 | 0.0362 | MsG0380015606.01 |
| GO:0016174 | MF | NAD(P)H oxidase H2O2-forming activity | 1 | 0.076923 | 0.0362 | MsG0480023541.01 |
| GO:0046857 | MF | oxidoreductase activity, acting on other nitrogenous compounds as donors, with NAD or NADP as acceptor | 1 | 0.071429 | 0.0390 | MsG0380015606.01 |
| GO:0030976 | MF | thiamine pyrophosphate binding | 1 | 0.071429 | 0.0390 | MsG0080048597.01 |
| GO:0080032 | MF | methyl jasmonate esterase activity | 1 | 0.066667 | 0.0417 | MsG0080048387.01 |
| GO:0080031 | MF | methyl salicylate esterase activity | 1 | 0.066667 | 0.0417 | MsG0080048387.01 |
| GO:0004743 | MF | pyruvate kinase activity | 1 | 0.066667 | 0.0417 | MsG0780041062.01 |
| GO:0030955 | MF | potassium ion binding | 1 | 0.066667 | 0.0417 | MsG0780041062.01 |
| GO:0016661 | MF | oxidoreductase activity, acting on other nitrogenous compounds as donors | 1 | 0.066667 | 0.0417 | MsG0380015606.01 |
| GO:0015120 | MF | phosphoglycerate transmembrane transporter activity | 1 | 0.0625 | 0.0444 | MsG0880046325.01 |
| GO:0015038 | MF | glutathione disulfide oxidoreductase activity | 1 | 0.0625 | 0.0444 | MsG0180004941.01 |
| GO:0008878 | MF | glucose-1-phosphate adenylyltransferase activity | 1 | 0.0625 | 0.0444 | MsG0380016036.01 |
| GO:0016701 | MF | oxidoreductase activity, acting on single donors with incorporation of molecular oxygen | 2 | 0.016529 | 0.0466 | MsG0880042633.01;MsG0180004203.01 |
| GO:0050505 | MF | hydroquinone glucosyltransferase activity | 1 | 0.058824 | 0.04718 | MsG0780038116.01 |
| GO:0071949 | MF | FAD binding | 2 | 0.016129 | 0.0487 | MsG0380015606.01;MsG0380011901.01 |
