# Supplementary material for: Analysis of physiological characteristics and gene co-expression networks in Medicago sativa roots under low-temperature stress
Source: Front Plant Sci. 2025 Aug 25;16:1597949. doi: 10.3389/fpls.2025.1597949 (PMC12415042; doi:10.3389/fpls.2025.1597949)
Supplement: Supplementary file 5 [file Table4.docx]

**KEGG pathway enrichment analysis of differential expressed genes in the root of Longmu801_vs_ Sardi alfalfa**

| **Pathway ID** | **Description** | **Number** | **Rich factor** | **P-value** | **First category** | **Second category** | **Gene ID** |
| --- | --- | --- | --- | --- | --- | --- | --- |
| map00940 | Phenylpropanoid biosynthesis | 5 | 0.020161 | 0.00132 | Metabolism | Biosynthesis of other secondary metabolites | MsG0280010428.01;MsG0780040942.01;MsG0780040943.01;MsG0580029291.01;MsG0780040945.01 |
| map00480 | Glutathione metabolism | 4 | 0.022346 | 0.00284 | Metabolism | Metabolism of other amino acids | MsG0380015008.01;MsG0480023109.01;MsG0380015009.01;MsG0180004941.01 |
| map00360 | Phenylalanine metabolism | 3 | 0.050847 | 0.00096 | Metabolism | Amino acid metabolism | MsG0780040942.01;MsG0780040943.01;MsG0780040945.01 |
| map04016 | MAPK signaling pathway - plant | 4 | 0.015748 | 0.00972 | Environmental Information Processing | Signal transduction | MsG0080048230.01;MsG0480023541.01;MsG0580025532.01;MsG0580024347.01 |
| map04075 | Plant hormone signal transduction | 5 | 0.011848 | 0.01246 | Environmental Information Processing | Signal transduction | MsG0780039188.01;MsG0880042776.01;MsG0080048230.01;MsG0580025532.01;MsG0580024347.01 |
| map00520 | Amino sugar and nucleotide sugar metabolism | 4 | 0.016064 | 0.00908 | Metabolism | Carbohydrate metabolism | MsG0280007789.01;MsG0380016036.01;MsG0780036078.01;MsG0280011136.01 |
| map00965 | Betalain biosynthesis | 1 | 0.1 | 0.03240 | Metabolism | Biosynthesis of other secondary metabolites | MsG0180004203.01 |
| map00966 | Glucosinolate biosynthesis | 1 | 0.076923 | 0.04192 | Metabolism | Biosynthesis of other secondary metabolites | MsG0580025858.01 |
| map00999 | Biosynthesis of various plant secondary metabolites | 2 | 0.018018 | 0.05165 | Metabolism | Biosynthesis of other secondary metabolites | MsG0380012889.01;MsG0880045441.01 |
| map00591 | Linoleic acid metabolism | 1 | 0.021277 | 0.14355 | Metabolism | Lipid metabolism | MsG0880042633.01 |
| map04712 | Circadian rhythm - plant | 1 | 0.013889 | 0.21145 | Organismal Systems | Environmental adaptation | MsG0180005357.01 |
| map00400 | Phenylalanine, tyrosine and tryptophan biosynthesis | 1 | 0.018868 | 0.16035 | Metabolism | Amino acid metabolism | MsG0480020595.01 |
| map00908 | Zeatin biosynthesis | 1 | 0.02439 | 0.12642 | Metabolism | Metabolism of terpenoids and polyketides | MsG0780041023.01 |
| map00040 | Pentose and glucuronate interconversions | 2 | 0.007752 | 0.20814 | Metabolism | Carbohydrate metabolism | MsG0780041316.01;MsG0880042836.01 |
| map00460 | Cyanoamino acid metabolism | 1 | 0.009615 | 0.29067 | Metabolism | Metabolism of other amino acids | MsG0380012889.01 |
| map00910 | Nitrogen metabolism | 1 | 0.021739 | 0.14072 | Metabolism | Energy metabolism | MsG0380015606.01 |
| map00073 | Cutin, suberine and wax biosynthesis | 1 | 0.015873 | 0.18764 | Metabolism | Lipid metabolism | MsG0180001149.01 |
| map00062 | Fatty acid elongation | 1 | 0.016949 | 0.17683 | Metabolism | Lipid metabolism | MsG0580024546.01 |
| map00941 | Flavonoid biosynthesis | 1 | 0.009901 | 0.28359 | Metabolism | Biosynthesis of other secondary metabolites | MsG0180005357.01 |
| map00960 | Tropane, piperidine and pyridine alkaloid biosynthesis | 1 | 0.014706 | 0.20095 | Metabolism | Biosynthesis of other secondary metabolites | MsG0180005357.01 |
| map00010 | Glycolysis / Gluconeogenesis | 2 | 0.010929 | 0.12156 | Metabolism | Carbohydrate metabolism | MsG0080048597.01;MsG0780041062.01 |
| map00500 | Starch and sucrose metabolism | 2 | 0.006873 | 0.24815 | Metabolism | Carbohydrate metabolism | MsG0380012889.01;MsG0380016036.01 |
| map00051 | Fructose and mannose metabolism | 1 | 0.010309 | 0.27404 | Metabolism | Carbohydrate metabolism | MsG0780036078.01 |
| map00592 | alpha-Linolenic acid metabolism | 1 | 0.010309 | 0.27404 | Metabolism | Lipid metabolism | MsG0580024178.01 |
| map00380 | Tryptophan metabolism | 1 | 0.010753 | 0.26436 | Metabolism | Amino acid metabolism | MsG0580025858.01 |
| map03015 | mRNA surveillance pathway | 1 | 0.005882 | 0.43016 | Genetic Information Processing | Translation | MsG0280009574.01 |
| map04626 | Plant-pathogen interaction | 2 | 0.004525 | 0.42847 | Organismal Systems | Environmental adaptation | MsG0280006794.01;MsG0480023541.01 |
| map02010 | ABC transporters | 1 | 0.00625 | 0.41090 | Environmental Information Processing | Membrane transport | MsG0180004752.01 |
| map00620 | Pyruvate metabolism | 1 | 0.00625 | 0.41090 | Metabolism | Carbohydrate metabolism | MsG0780041062.01 |
| map00195 | Photosynthesis | 1 | 0.006494 | 0.39905 | Metabolism | Energy metabolism | MsG0880045275.01 |
| map00564 | Glycerophospholipid metabolism | 1 | 0.006536 | 0.39705 | Metabolism | Lipid metabolism | MsG0180005354.01 |
| map04144 | Endocytosis | 1 | 0.00271 | 0.70694 | Cellular Processes | Transport and catabolism | MsG0380015761.01 |
| map03040 | Spliceosome | 1 | 0.002358 | 0.75645 | Genetic Information Processing | Transcription | MsG0380015761.01 |
| map04141 | Protein processing in endoplasmic reticulum | 1 | 0.001992 | 0.81286 | Genetic Information Processing | Folding, sorting and degradation | MsG0380015761.01 |
